# Tracking of adult males and females across a migratory divide: migration strategies of a western baltic common tern (*Sterna hirundo*) population

**DOI:** 10.1186/s40462-026-00666-6

**Published:** 2026-05-30

**Authors:** Simon Piro, Eva Stöbbe, Matthias H. Weissensteine, Angela Schmitz Ornés

**Affiliations:** 1 AG Vogelwarte, Zoological Institute and Museum, University of Greifswald, 17489 Greifswald, Germany; 2 Institute of Avian Research “Vogelwarte Helgoland”, 26386 Wilhelmshaven, Germany

**Keywords:** Light-level geolocation, Timing of migration, Duration of migration, Eastern African flyway

## Abstract

**Background:**

European Common Terns’ migration routes along the eastern Atlantic flyway and wintering areas in western and southern Africa were identified in ringing studies. Recent tracking studies revealed a migratory divide and the use of an eastern African migration route and wintering areas in the Mozambique Channel.

**Methods:**

71 Common Terns breeding in a colony at the German Baltic coast were tracked with light-level geolocators. Linear Models were used to test influence of sex, year, migration route, and wintering area on timing of migration, and duration of migration and stopovers.

**Results:**

A total of 58 individuals used the western African migration route, while 13 individuals used the eastern route. 45 individuals wintered at the southern African coast, while 12 individuals wintered in the Gulf of Guinea, and 13 individuals wintered in the Mozambique channel. Migration route and wintering area were identified as the most important factors influencing the duration of migration and stopovers, with birds using the eastern route showing significantly longer stopovers and duration of migration. Sex was identified to especially impact the start of migration, with females starting both autumn and spring migration significantly earlier than males. Arrival in the breeding area was only affected by the year, but this might be an effect of very low sample size in some years. Birds wintering in western Africa showed a tendency to a longer spring migration, while those wintering in eastern Africa tended to longer autumn migration.

**Conclusion:**

The eastern African migration route and wintering area seem to be much more common than ringing had indicated. Route and sex explain a substantial part of the observed variation in timing and duration of migration, but many factors such as annual variation in weather, wind, food availability as well as individual variation in fitness and breeding success are likely to affect timing of migration and duration of migration and stopovers. Comparison of duration of autumn and spring migration indicates that contradicting results reported in tracking studies from different European breeding areas might be explained by wintering area rather than breeding area, but are pronounced in birds with shorter migration distance.

**Supplementary Information:**

The online version contains supplementary material available at 10.1186/s40462-026-00666-6.

## Background

Annual migration is a widespread phenomenon in animals, found not only in birds, but also in mammals, fish, reptiles, marine invertebrates, and land insects [1, 2]. It is usually driven by seasonal fluctuation in food availability [1, 3], and allows areal expansion and the exploitation of seasonal resource peaks in areas which are not suitable for year-round use [2, 4]. However, especially long-distance migrants depend on a linked chain of stopover sites to rest and rebuild energy stores, which are crucial for them to complete their annual migration [5]). In a fast changing world, understanding migratory flyways and stopover sites is essential for the conservation of migratory species [6], as threats to a single essential site in the migratory cycle might drive rapid population declines [7]. In the case of the European Common Tern (*Sterna hirundo*), migration routes, stopover sites, and wintering areas were thought to be well studied based on numerous ringing studies, which show that they use the East Atlantic flyway to reach their wintering areas at the African coast and suggested a ‘leap frog’ migration, with birds from northern and eastern Europe migrating further south than birds from western, central, and southern Europe [8–12]. Main wintering area of Common Terns breeding in western and central Europe as well as Great Britain were found to be in western Africa at the coasts between Western Sahara and Nigeria [8, 13, 14], while main wintering areas of birds breeding in Scandinavia and eastern Europe, including the German Baltic population, were found to be at the coasts of Namibia and South Africa [8–10, 15–17]. Wintering Common Terns found at the eastern African coast were presumed to belong to the subspecies *S.h. tibetana*, breeding in the central Asian mountains [18]. Although Common Terns ringed in Germany and Austria were found at the eastern African coast in Tanzania, it was presumed they had circumvented the Cape of Good hope and reached Tanzania on the East Atlantic flyway [18]. Even when the first German ringed Common Tern was recaptured in Israel, it was considered a rare exception [19]. This view on European Common Tern migration only recently changed, when tracking studies on birds from Croatia, Hungary and Germany revealed a migratory divide and the use of the Eastern African flyway leading to wintering areas in south eastern Africa [4, 20, 21]. Migratory divides are found in numerous European bird species and often develop out of the necessity to avoid geographical barriers, or as a result of separated breeding populations developing separated resting or wintering areas [22]. Due to spatial differences in food availability, climate and threats, the use of different migration routes and wintering areas can directly affect the survival of birds during migration and wintering, as well as their breeding performance through carry-over effects [23, 24].

In a first tracking study, we [4] discovered that Common Terns breeding in a colony on an island called Riether Werder, located at the northeastern German coast use both, the eastern and western African flyways and winter in three main wintering areas: One located in western Africa in the Gulf of Guinea, one located at the coast of Namibia and South Africa, and one at the eastern African coast in the Mozambique Channel. Although the study only contained 23 individuals, we discovered interesting differences in the timing and duration of migration of birds using different migration routes. Both migration phases lasted significantly longer in birds using the eastern migration route, as birds on the eastern route spent much more time at stopover sites than birds using the western route. To compensate for their longer migration, birds using the eastern route started spring migration significantly earlier, and we found no difference in the arrival time at the breeding area. In a second study [25], no assortative mating was found for either migratory route or wintering area, as birds using both flyways and all three wintering areas paired randomly, which might be explained by a lack of difference in the arrival date at the colony between the groups. We presumed, that migratory direction and wintering sites might be passed to young terns via social learning, either by joining the parents or migratory flocks of conspecifics [25]. If migratory phenotypes are passed on by a parent, it seems more likely that it is passed from father to young, as female Common Terns tend to leave the colony earlier [26, 27] and males provide the majority of post-fledging care [27, 28].

In this study, we analyze light level geolocation tracking data of Common Terns breeding in the Riether Werder colony after several more years of tracking, and with a much larger sample size. Building on our previous results we explore different models examining how timing and duration of migration and stopover behavior were influenced by migration route and wintering area, and also incorporated sex into our models. To address annual variation in external conditions like weather, wind, or food abundance we also included the effect of the study year into the models.

We hypothesize that timing and duration of autumn migration should be influenced by sex, as females should start their autumn migration earlier than males, independently from route or wintering area. If so, we also expect them to arrive earlier in their wintering areas, unless they spend more time at stopover areas. In that case, we also expect them to have a longer duration of autumn migration than males.

In case of spring migration, we expect it to be mainly influenced by route and wintering area, as birds on the eastern route wintering in eastern Africa compensate for their longer migration by starting spring migration earlier [4].

Regarding the duration of migration phases, contradicting results have been reported in previous tracking studies in Common Terns breeding in north western Germany [29], North America [30], Croatia, and Hungary [20]. In Piro and Schmitz Ornés [4] we found no differences in the duration of migration of birds tagged with geolocators in the Riether Werder colony (north eastern Germany), neither when tested for all tracked birds nor when tested separately for birds using the eastern or the western route. In the present study with a higher sample size and individuals using different routes, we hypothesize that, if there is a difference in duration between spring and autumn migration, birds on the western route should show a longer spring migration, similar to the birds tracked by Becker et al. [29] while the ones on the eastern route, similar to the birds tracked by Kralj et al. [20], should show a longer autumn migration.

## Methods

Study area and deployment of loggers:

Riether Werder (54°42’ N, 014°16’E) is an 82 ha Island in the German part of the Szczecin Lagoon. It is part of the special protected area *Kleines Haff, Neuwarper See und Riether Werder* (DE 2250–471). With 10.000–12.000 pairs, it hosted the largest Black-headed gull colony in Germany [31]. Common Terns use the shelter of this colony and form a smaller colony between the gulls, using the sandy area at the dike as a nesting site. Between 2019 and 2023, a total of 120 adult Common Terns of the Riether Werder colony were fitted with light level geolocators (Intigeo-W65A9-SEA, Migrate Technology Ltd) mounted to plastic leg rings (2019: *n* = 40, 2020: *n* = 23, 2022: *n* = 40, 2023: *n* = 17). For details about programming of loggers see Additional file 1.

A total of 79 of the birds tagged with a logger were recaptured. As some loggers failed before the bird started migration (*n* = 2) or the birds returned without loggers (*n* = 6) a total of 71 individuals were tracked. Two of these birds could be tracked for two full years resulting in a total of 73 tracks, while most of the individuals (*n* = 66) only for one year. Five birds were tracked for less than a year, as the loggers stopped recording at some point during the migratory cycle. While 71 of the 79 birds were recaptured at Riether Werder, one of the birds was recaptured 23 km south from the island in a small colony at Lake Krugsdorf (53°32’ N, 14°5’ E) while a total of seven birds were recaptured in the Polish part of the Sczcecin Lagoon, 16 km west from Riether Werder, in the colony on Śmięcka Island (53°45’ N, 14°13’ E). For a summary of timing and duration of migration of all birds see Additional file 2.

Sexing of individuals:

Breast feathers and blood samples were used to genetically determine the sex of the birds. In 2019, three breast feathers were collected of each bird for genetic analyses [32]. Feather samples were stored dry and DNA was extracted using the DNeasy Blood and Tissue Kit (Qiagen, Germany) following the manufacturers protocol. To determine sex from feather samples universal P2/P8 primers [33] were used. PCR reactions were performed using a GoTaq Hot Start Green Master Mix Kit (Promega), according to the manufacturers protocol. The amplified PCR fragments were checked with electrophoresis on a 1.5% agarose gel.

Since 2020, blood samples have been collected from the ulnar vein of most of the birds. Blood samples were stored in 95% EtOH at −80 °C and sex of individuals was determined using whole-genome sequencing (Stoebbe et al. unpublished data) by comparing the read depth of chromosome Z to that of an autosome. The ratio of read depth for chromosome Z relative to an autosome is approximately 1 in males (two copies of both the Z chromosome and the autosome) but approximately 0.5 in females (one copy of the Z chromosome and two copies of the autosome).

Analysis of tracking data:

To analyze migration routes and wintering areas, light level analyses were conducted in R (v4.0.0 [34], using R-Studio (v4.0.2 [35], according to the online supplementary manual of Lisovski et al. [36]. The package BAStag (v0.1.3 [37] was used for twilight determination and FLightR (v0.5.1 [38] for the analysis (Additional file 1). Stopovers were defined as stationary periods where an individual had spent at least 10 twilights (5 days) in a given area, as Common Terns are thought to use stopover sites for more than 5 days [20, 29]. Duration of migration was defined as the time between departure from the Szczecin Lagoon or, in birds showing post-breeding dispersal (movements resulting in stopovers of ≥20 days which were still ≥ 50°N, for details see [4]), departure from the area to which they had dispersed, and arrival at the wintering area and vice versa.

According to their migratory direction, birds were divided into those using the western route (migrating in south western direction) and the eastern route (migrating in south eastern direction). Three main wintering areas were defined at the western African coast (north of the Equator, between Western Sahara and Nigeria), the southern African coast (Namibia and western South Africa) and the eastern African coast (in the Mozambique Channel) in accordance with our previous publications [4, 25].

Analyses of timing and duration of migration:

For the analyses we separated the birds into the following groups according to their migratory direction and wintering grounds: e.eastern: using the eastern flyway and wintering in eastern Africa, s.western: using the western flyway and wintering in southern Africa, w.western: using the western flyway and wintering in western Africa, s.eastern: using the eastern flyway and wintering in southern Africa, e.western: using the western flyway and wintering in south eastern Africa. As the groups s.eastern and e.western each only consisted of a single bird they were excluded from further analyses. For the two individuals which were tracked twice, only the 2023–2024 migration cycle was used in the analyses.

Using complete tracks of birds with known sex (*n* = 55) in an explorative analysis, we built linear models (LM) to test the influence of the categorical variables sex, migration cycle (year) and the combined variable of migration route and wintering area on the response variables start and duration of autumn migration, days spent at stopover sites during autumn migration, arrival at the wintering area, start and duration of spring migration, days spent at stopover sites during spring migration and arrival at the breeding area. We selected the best combination of variables for each full model using the function *step* for a backward stepwise regression getting the best model, and compared these two with a null model (without any of the three fixed factors) using the function *compare_performance* from the “performance” package (v0.15.3 [39], in R (4.5.2 [40]. Model fits and checking of residuals were done with “performance” package as well. Function *summ* was used from the package “jtools” (v2.3.0 [41], to create the model-summary table. Pairwise contrast was done using the function *emmeans* of the package “emmeans” (v2.0.0 [42]. Effect plots were created with “effects” (v4.2.4 [43], “ggplot2” (v4.0.1 [44], and gridExtra (v2.3 [45].

Using Wilcoxon signed-rank tests with Holm-Bonferroni adjustment we also tested for differences between the duration of autumn and spring migration for all birds with complete tracking cycles (*n* = 64), as well as separated for the e.eastern group (*n* = 11), w.western group (*n* = 11), and s.western group (*n* = 42).

## Results

**Fig. 1:**
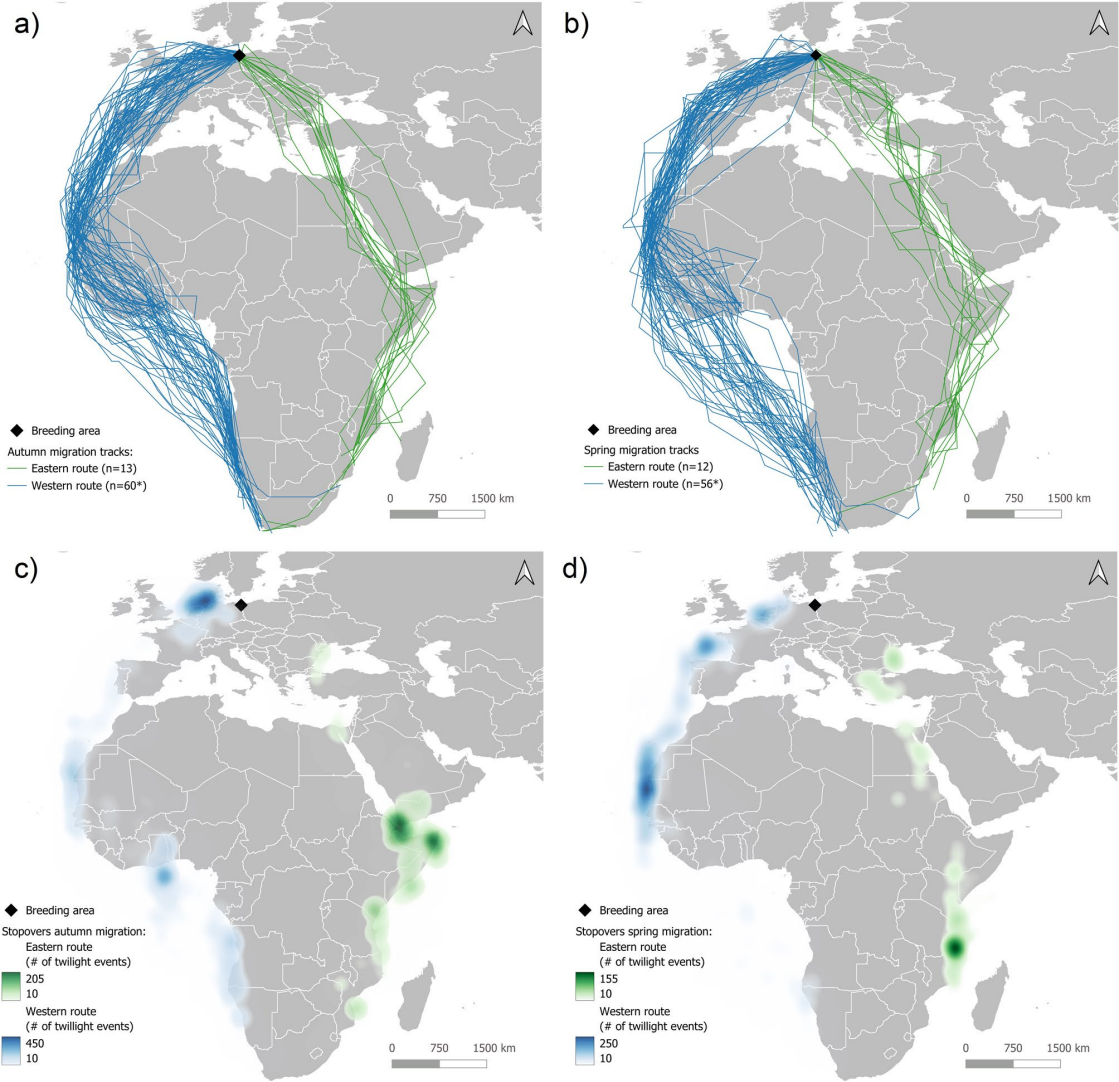
Autumn migration tracks (**a**), spring migration tracks (**b**) and stopover sites plotted as heat maps for autumn migration (**c**) and spring migration (**d**) of 71 Common Terns tagged with light-level geolocators in the colony on Riether Werder. *two individuals were tracked twice. Map: geoportal of the European commission (EUROSTAT): https://ec.Europa.eu/eurostat/web/gisco/geodata/reference-data/administrative-unitsstatistical-units/countries

Routes, stopover sites, and wintering areas:

Figure 1 shows the tracks of autumn (Fig. 1a) and spring migration (Fig. 1b), as well as the stopover sites during both periods (Fig. 1c and d). A total of 58 (81.7% of all tracked) birds used the western migration route, following the East Atlantic flyway through the Wadden Sea, along the coasts of France, Portugal and Spain. After crossing the Strait of Gibraltar they followed the western African coast to their main wintering areas in western and southern Africa (Fig. 1a).

During autumn migration, most important stopover regions on the western route were in the Wadden Sea and at the western African coast (Senegal and Mauritania, Fig. 1c). For birds migrating further south, also the Gulf of Guinea was identified as an important stopover area, where 11 birds (19.6% of the birds using the western route and 15.9% of all birds) wintered.

**Fig. 2:**
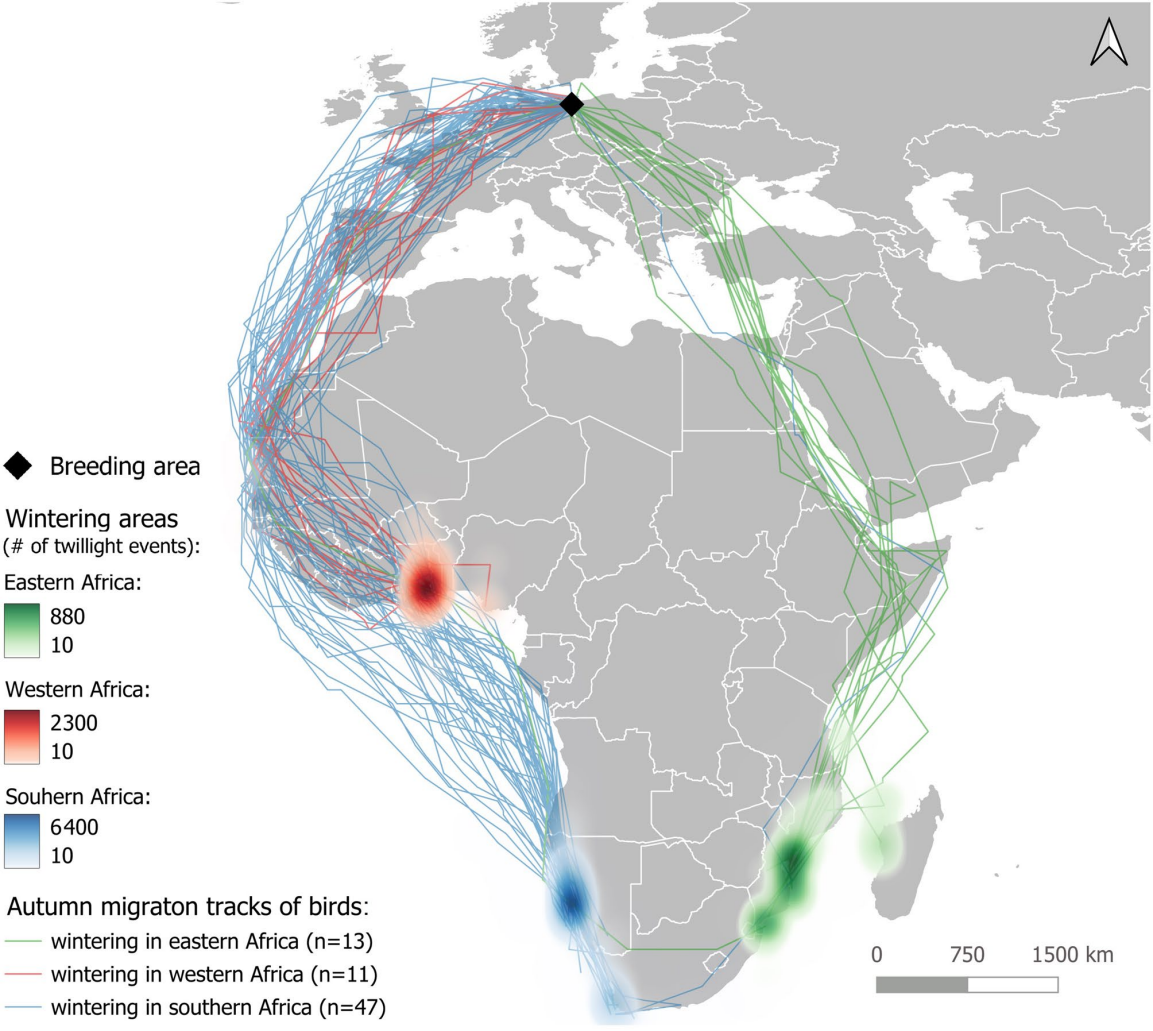
Autumn migration tracks and wintering areas plotted as heatmaps of 71 Common Terns tagged with light level geolocators in the Riether Werder colony. Map: geoportal of the European commission (EUROSTAT): https://ec.Europa.eu/eurostat/web/gisco/geodata/reference-data/administrative-unitsstatistical-units/countries

With a total of 45 individuals (65.2%), most of the birds wintered in southern Africa at the coasts of Namibia and South Africa (Fig. 2). 44 (78.6%) of the birds using the western route were joined by one bird from the eastern Route, which after rounding the Cape of good Hope wintered in the area around Cape Town (South Africa, see Fig. 2). The most important wintering area for Common Terns from the Riether Werder colony was found in Walvis Bay near Swakopmund (Namibia, Fig. 2). Two loggers stopped working while the birds were still on autumn migration. While they could be assigned to the western migration route, their wintering area remains unknown. One individual using the western route rounded the Cape of good Hope as well and wintered in the southern Mozambique Channel (Fig. 2). The two individuals that were tracked for two successive years both used the western route and wintered in southern Africa in both years.

**Fig. 3:**
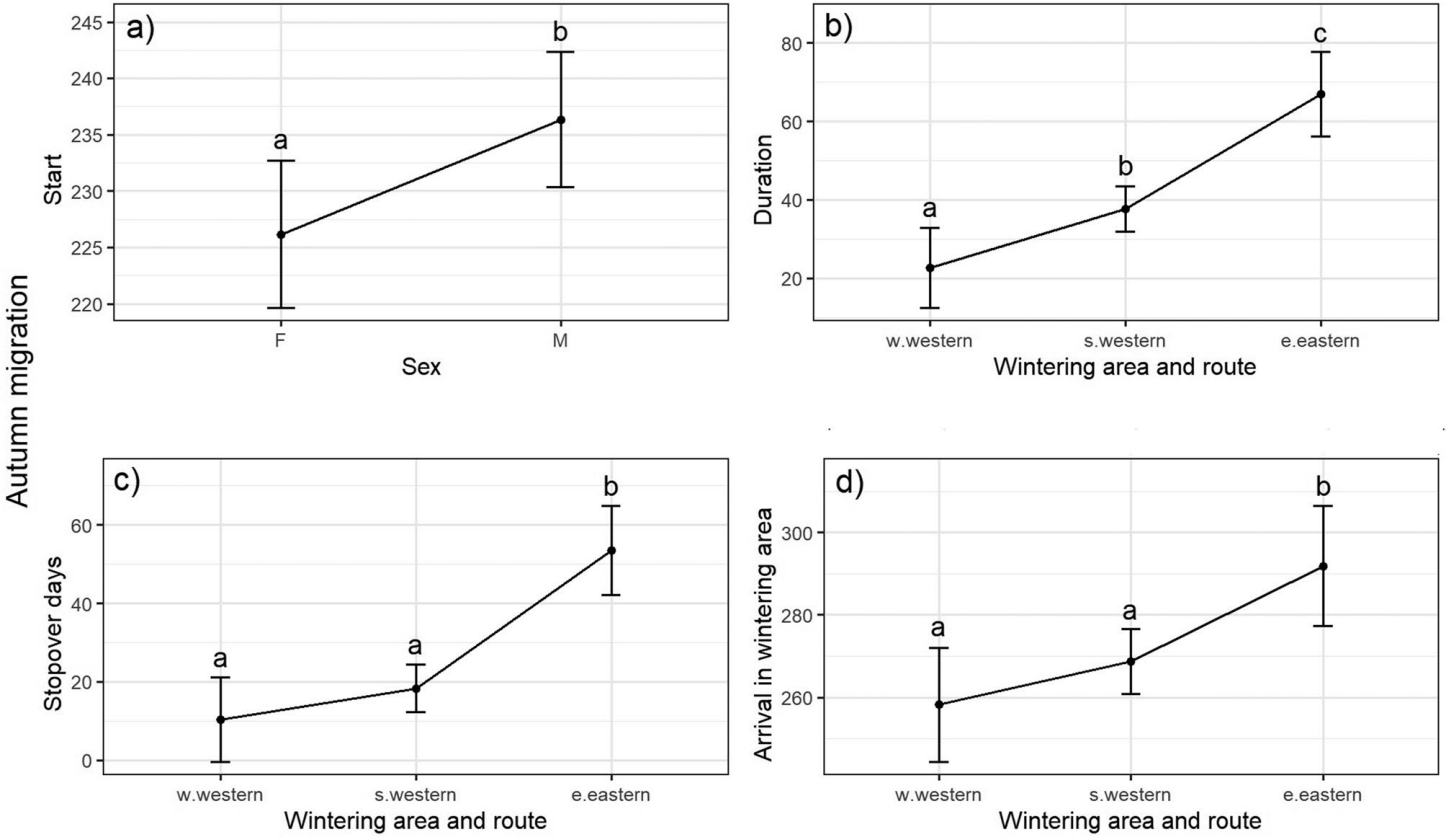
Effect plots showing the effect of sex (30 males and 25 females) and the combined variable of migration route and wintering area (11 individuals taking the western route and wintering in western Africa, 34 on the western route wintering in southern Africa, and 10 individuals taking the eastern route and wintering in eastern Africa) on a) start and b) duration of autumn migration, c) number of days spent at stopover sites, and d) arrival in the wintering area. Migration cycle had no significant effect

A total of 13 (18.3%) birds used the eastern migration route. They either crossed central Europe directly heading to the eastern Mediterranean Sea or made a detour to the Black Sea, usually associated with a short stopover at its western coast (Fig. 1a and c). After crossing or rounding the eastern Mediterranean Sea, they followed the Red Sea south. The southern red Sea and Gulf of Aden was by far the most important stopover site during autumn migration (Fig. 1c). Following the eastern African coast, they reached their main wintering area in the southern Mozambique channel (Fig. 2).

A total of 13 birds (1.8% of the birds using the western route, 92.3% of the birds using the eastern Route, 18.8% of all tagged birds) wintered at the eastern African coast, in the Mozambique Channel, where 11 individuals wintered at the Mozambique coast, while two wintered at Madagascar (Fig. 2). One individual using the eastern route rounded the Cape of Good hope, wintering in the area around Cape Town (south Africa).

On both flyways, routes of spring migration were very similar to autumn migration (Fig. 1a and b). Stopovers however varied between migration phases. On the western route, the Gulf of Guinea, which was an important stopover site of birds wintering in southern Africa during autumn migration, was not used as stopover site during spring migration (Fig. 1b and d). Another important stopover area was identified in the Bay of Biscay that was only used during spring migration. Stopover sites on the eastern Route also differ between autumn and spring migration. The southern Red Sea and Gulf of Aden, which was the most important stopover area during autumn migration, was not used during spring migration. However, an important stopover area during spring migration was in the northern Mozambique channel, while other stopover areas were located in the northern Red Sea, the eastern Mediterranean Sea, and the western coast of the Black sea (Fig. 1b and d).

Timing and duration of migration:

The models for autumn migration show that sex has a significant effect on starting (Fig. 3a, Additional file 3), with females starting on average 10.2 days earlier than males (SE = 4.55, *t*_(53)_ = 2.24, *p* = 0.030, *n* = 55). However, the overall model explained only a small portion of the variance (R^2^ = 0.090, adj. R^2^ = 0.074). There seems to be no evidence for a further effect of sex on the rest of autumn migration. All, duration, stopover days, and arrival in the wintering areas were significantly affected by route and wintering area. Individuals wintering in eastern Africa (eastern route) were on migration longer than their conspecifics on the western route: 44.3 days (SE = 7.42, t_(52)_ = −5.97, *p* < 0.001, *n* = 55) longer than those wintering in western Africa, and 29.2 days (SE = 6.11, t_(52)_ = −4.79, *p* < 0.001, *n* = 55) longer than those wintering in southern Africa (Fig. 3b, Additional file 3). On the western route, birds wintering in southern Africa migrated 15.0 days (SE = 5.89, t_(52)_ = −2.55, *p* = 0.036, *n* = 55) longer than those wintering in western Africa (Fig. 3b, Additional file 3). Similarly, birds wintering in eastern Africa (eastern route) spent more time on stopover sites than those on the western route, 43.2 days (SE = 7.78, t_(52)_ = −5.56, *p* < 0.001, *n* = 55) than those wintering in western Africa and 35.2 days (SE = 6.40, t_(52)_ = −5.49, *p* < 0.001, *n* = 55) than those wintering in southern Africa (Fig. 3c, Additional file 3).

Birds using the eastern route also arrived later in their wintering area than their conspecifics on the western route, 33.6 days (SE = 10.00, t_(52)_ = −3.35, *p* = 0.004, *n* = 55) later than those wintering in western Africa, and 23.2 (SE = 8.25, t_(52)_ = −2.81, *p* = 0.019, *n* = 55) days later than those wintering in southern Africa (Fig. 3d, Additional file 3).

**Fig. 4:**
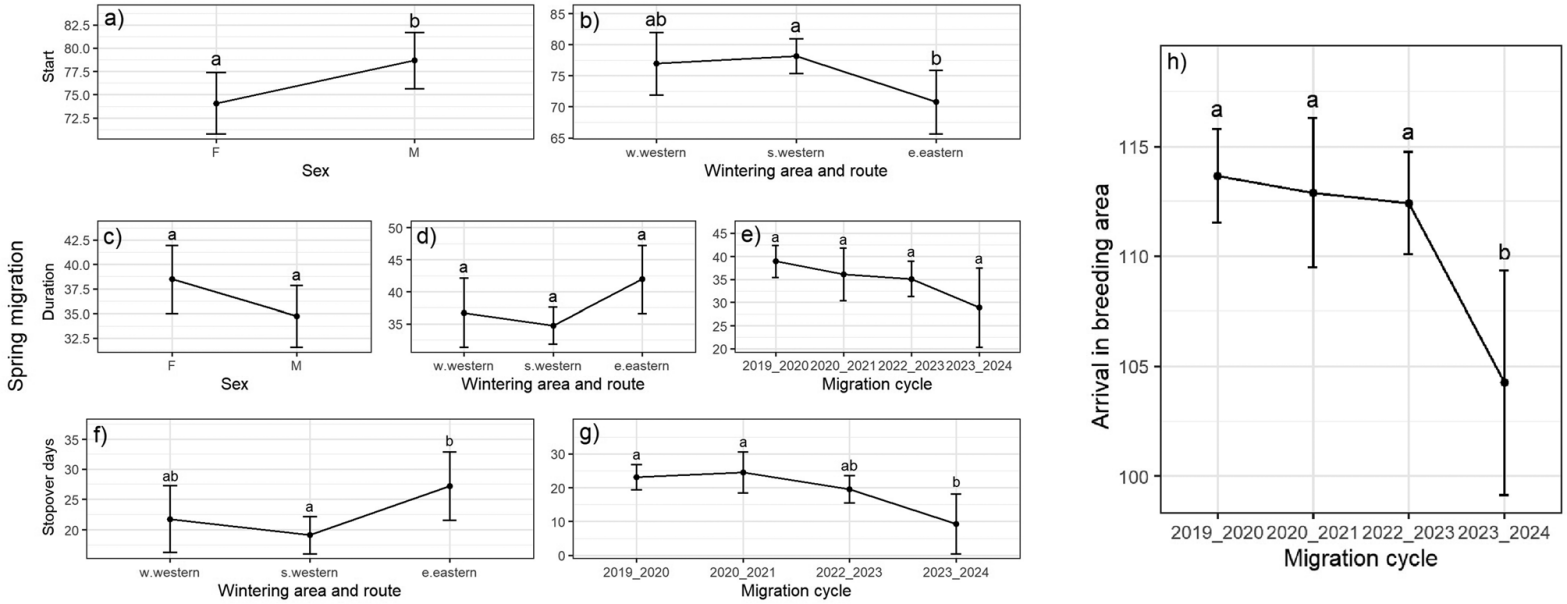
Effect plots showing the effect of sex (30 males and 25 females), migration cycle (23 individuals in 2019_2020, 9 in 2020_2021, 19 in 2022_2023, and 4 in 2023_2024), and the combined variable of migration route and wintering area (11 individuals taking the western route and wintering in west, 34 on the western route wintering in south, and 10 individuals taking the eastern route and wintering in east) on start and duration of autumn migration, number of days spent at stopover sites, and arrival in the wintering area

For spring migration, there is an influence of route and wintering area, however, sex and year of migration also seem to play a role. The best model for start of spring migration included both sex and the combination of route and wintering area as predictors and showed slightly stronger overall fit (R^2^ = 0.191, adj. R^2^ = 0.143) than the model for start of autumn migration. Females again started earlier than males (4.6 days, SE = 2.07, t_(51)_ = 2.21, *p* = 0.031, *n* = 55, Fig 4a, Additional file 3), and individuals following the eastern route and wintering in eastern Africa started significant earlier than the ones on the western route wintering in southern Africa (7.4 days, SE = 2.90, t_(51)_ = 2.56, *p* = 0.036, *n* = 55, Fig. 4b, Additional file 3). Sex and the combination of route and wintering area also tended to influence the duration of spring migration. Duration of migration was 3.8 days shorter in males than in females (SE = 2.02, t_(48)_ = −1.87, *p* = 0.068, *n* = 55, Fig. 4c, Additional file 3), and individuals on the eastern route wintering in eastern Africa migrated between 5.2 (SE = 3.81, t_(48)_ = −1.37, *p* = 0.367, *n* = 55) and 7.2 (SE = 3.00, t_(48)_ = −2.41, *p* = 0.051, *n* = 55) days longer than those using the western route and wintering in western and southern Africa, respectively (Fig. 4d, Additional file 3). Additionally, although years of migration seem to have an effect on the duration of spring migration, due to the large uncertainty caused by the small sample size per year (especially 2024), there is no evidence of an effect of years when conservative multiple-comparison correction with Tukey is done (Fig. 4e, Additional file 3).

The days spent at stopover sites during spring migration seem to be affected by route and wintering area and migration cycle, with 8.1 days (SE = 3.18, t_(49)_ = −2.53, *p* = 0.038, *n* = 55) longer stopovers for birds on the eastern route wintering eastern Africa compared to those wintering in southern Africa (Fig. 4f, Additional file 3). Only the spring migration in 2024 seems to show shorter stopover times as in previous years (Fig. 4g, Additional file 3).

For the arrival in the breeding area only year of the migration cycle indicates a significance, with birds arriving earlier in 2024 than in previous years (Fig. 4h, Additional file 3).

General differences between autumn and spring migration:

When tested for all birds, there was no evidence of a difference between the duration of autumn and spring migration (V = 1281.5, *p* = 0.262, adjusted *p* = 0.469, *n* = 66, Additional file 4). When separately tested according to wintering group (associated of course to route), we found tendencies (although not significant) for longer spring migration in birds wintering in western Africa (V = 10, *p* = 0.045, adjusted *p* = 0.181, *n* = 11, Additional file 4), and longer autumn migration for birds wintering in eastern Africa (V = 52, *p* = 0.100, adjusted *p* = 0.299, *n* = 11, Additional file 4).

## Discussion

### Routes, stopover sites, and wintering areas:

The majority of the Common Terns from Riether Werder used the eastern Atlantic flyway along the western European and African coast. Their migratory routes, as well as stopover sites and wintering areas, were quite similar to the ones documented for tracked Common Terns from the German Wadden Sea [26, 29], although a much larger proportion of the Riether Werder birds wintered in southern Africa. A recent tracking study from southern Sweden revealed that Swedish birds also use the western route, and winter at the Namibian and South African coast [46]. Common Terns from Scandinavia and the Baltic migrating further south than western European ones corresponds with the results of previous ringing studies [8–10, 15–17], and support the hypothesis of ‘leap frog’ migration reported by other authors [8–12].

Interestingly, although breeding only about 250 km north from Riether Werder, the use of the eastern migration route could not be documented for tracked Common Terns breeding in southern Sweden [46]. However, a recent ringing project from Israel indicates that birds using the eastern route may breed in larger numbers in eastern Europe [47].

Our results suggesting that Walvis Bay is the most common wintering area of Common Terns from Riether Werder agrees with Wearne and Underhill [48], who estimated that with hosting up to 15% of the whole flyway population, Walvis Bay is one of the most important wintering areas of western Palearctic Common Terns. Other wintering areas in southern and western Africa are in accordance with previous ringing studies [8–10, 15–17].

Contrary to the western route, the eastern route has not been described from ringing data yet. It had only recently been described based on tracking studies [4, 20, 21]. Only very few ringed Common Terns from Germany recovered in south eastern Africa [10, 16, 18], as well as some recent recoveries in Israel [19, 47, 49, 50], may have indicated the use of the eastern route. Although our tracking data showed that birds using the western route can cross the Cape of Good Hope and winter in the southern Mozambique Channel, the vast majority of our tracked birds that winter at the eastern African Coast reached their wintering area on the eastern route. Therefore, contrary to former assumptions made by Hume [18], it is likely that the birds from Germany recovered along the eastern African coast may have reached their destination on the eastern route.

The routes used by birds from Riether Werder along the eastern African coast are very similar to the ones documented for Hungarian and Croatian Common Terns [20, 21]. Interestingly, at Riether Werder birds using both migration routes breed in the same colony, and with each other [25], while in Croatia, birds using the eastern and birds using the western route breed in separated colonies [21].

As shown in a previous study [21], stopover sites and wintering areas along both routes are located in areas with high primary production, guaranteeing a high food abundance for Terns, as the availability of small fish, squids, and zooplankton, is directly related to the abundance of phytoplankton [51, 52]. In western and southern Africa, the high primary production in coastal waters is a result of strong upwelling currents [53, 54]. These upwelling currents steadily bring cold and nutrient rich water from the deep to surface layers, securing permanent high primary production, with the Benguela current upwelling system at the Namibian and western south African coast being one of the world’s most productive marine ecosystems [54].

Along the eastern African coast however, primary production is not driven by steady upwelling currents, but by Monsoons in the Red Sea and Gulf of Aden [55, 56], and eddy activity in the Mozambique channel [57], which bring cold and nutrient rich water to the surface. This results in an unsteady and uneven distribution of primary production through the year, which most likely is responsible for the differences in stopover sites during autumn and spring migration, as the birds will rest in areas with high primary production to refill. Although the sample size is too small for statistical analysis, the two birds tracked for two years agree with the results of Kürten et al. [26] showing a high repeatability of migration route and wintering area.

Timing and duration of migration:

Route and wintering area effect:

Although the sample sizes of birds wintering in eastern Africa (eastern route) and western Africa (western route) were relatively small, limiting the statistical strength of our models, the results show the consistent influence of both route and wintering area (combined) on duration of migration and stopovers. As already stated in a previous study [4], one of the most interesting differences between routes is the much longer duration of migration in the birds using the eastern migration route. Our results suggest that the longer duration of migration is a result of longer stopovers. These are most probably caused by a general lower food abundance on the eastern route [21] and peaks in primary production in the southern Red Sea and Gulf of Aden in August and November [55], prolonging migration especially during autumn migration. The linear models for duration of migration and days spent at stopover sites also were the best overall, each explaining about 42% of the variances during autumn migration, and 27 and 29% during spring migration. While birds using the eastern route arrive later in their wintering areas due to their longer migration, they seem to compensate for that by starting spring migration significantly earlier. Route and wintering area therefore was associated with arrival in the wintering area, and start of spring migration, but not with start of autumn migration and arrival in the breading area. Similar to our previous studies [4, 25], our results suggest that, regardless of migration route and wintering area, birds breeding in the Riether Werder colony arrive simultaneously in the breeding area. This lack of difference in arrival may be one of the key factors for the lack of assortative mating resulting in pairing of birds using different routes and wintering areas in the Riether Werder colony [25]. Early arrival, as soon as the conditions in the breeding area allow it, may be under selection, as the birds have to compete for high quality nest sites and partners, resulting in the observed simultaneous arrival between all groups.

Sex effect:

As we expected, females started migration earlier than males, not only during autumn migration, but also in spring. While there was no other evidence of sex related differences for the rest of the autumn migration, in spring, females spent a longer time on migration arriving at the same time as males back to the breeding area.

Females starting autumn migration earlier than males seems to be a widespread phenomenon in single-clutch shorebirds [58], and has been shown for Common Terns [26, 27, 59], Whiskered Terns (*Chlidonias hybrida* [60]), and Caspian Terns (*Hydroprogne caspia* [61]). This phenomenon possibly originates in a sexual conflict where earlier leaving females gain in terms of residual fitness over time [61, 62]. The female’s desertion often results in males taking the responsibilities for post-fledging parental care and in some species to guide the offspring during its first migration, as recently shown for Caspian Terns [61]. A similar form of guided migration might also lead to the passing of migratory direction and wintering area from father to offspring by social learning in Common Terns [25].

Female Common Terns also starting their spring migration earlier than males poses an interesting phenomenon, as in most species in spring, males migrate earlier and arrive in the breeding area before females [63]. Our results suggest that by starting earlier, females may profit from a slower migration. As egg production is costly in terms of required nutrients [64], females demand much higher energy during the egg laying season. While capital breeders use endogenous nutrients for egg production [65, 66], income breeders use locally derived exogenous nutrients [67]). Most species, however, are neither pure capital or income breeders [68, 69]. Although some Common Tern populations have been found to be income breeders [64, 70], Hobson et al. [71] found that Common Terns from Great Slave Lake (Canada) rely on endogenous reserves for egg production, which suggests that nutrient allocation may be a plastic trait within Common Terns, and that this philopatric species might adapt their form of nutrient allocation to the nutrient availability in their breeding area (Bond and Diamond [64]. Although it is unknown if Common Terns from the Riether Werder colony rely on endogenous reserves, it is likely that carry over effects from migration may affect their reproductive success, which might increase the fitness of females migrating slower compared to males.

If earlier starting and slower migrating females is common in other species may be hard to address. While the early arrival of males in the breeding area can be documented from observation alone, especially in birds with sexual dimorphism, tracking of individuals is needed to document start and duration of migration. Ongoing miniaturization of tracking devices might make it possible to address this question on a larger number of species in the near future.

Migration years and other effects:

As our tracking study was conducted over several years, weather and wind conditions might have varied between years, affecting start of migration, duration of migration and stopovers, as well as arrival in the wintering and breeding areas. Similarly, variation in food availability between years might influence the stopover behavior, especially on the eastern route, where primary production is not as steady and therefore less predictable as on the western route, and depends on monsoons and eddy activity [55–57]. Although we included the years of the tracked migration cycle into our models and indeed found effects on spring migration, the small sample sizes especially in the 2023–2024 migration cycle prevent us to make a strong argument in this regard. Additionally, individual aspects as physical condition, individual experience or breeding success and possible parental guidance of offspring most probably have huge impact on timing of migration and its duration as well as the duration of stopovers.

Comparison of autumn and spring migration:

In many bird species, autumn migration lasts longer than spring migration [72]. For the Common Tern however, contradicting results have been reported. While Becker et al. [29] showed a significant longer duration of spring migration for western German Common Terns, Nisbet et al. [30] and Kralj et al. [20] found the opposite for Common Terns breeding in Northern America, Croatia, and Hungary. Our previous study found no differences in the duration of migration phases, but our larger dataset now allowed testing our hypothesis for wintering areas, revealing at least a tendency to opposite patterns between groups with a longer autumn migration for birds using the eastern African flyway and wintering in eastern Africa, like in Kralj et al. [20], and longer spring migration for birds using the western route and wintering in western Africa like in Becker et al. [29], although the differences were less pronounced than in these studies. These results suggest, that differences between the duration of autumn and spring migration might be dependent on the wintering area rather than the breeding area, and might be more pronounced in birds with shorter distance between breeding and wintering area, such as birds studied by Becker et al. [29] and Kralj et al. [20].

The longer autumn migration in birds using the eastern route appears to be a result of the very long stopovers in the southern Red Sea and Gulf of Aden these birds do during autumn migration, but not during spring migration. These long stopovers might be linked to peaks in primary production in August and November in this region [55].

Becker et al. [29] assumed longer spring migration along the western route to be a consequence of prevailing winds, rotating clockwise in the North Atlantic and offering tailwind during autumn migration, but headwind during spring migration [73]. However, this does not explain why especially the birds wintering further south seem to be unaffected by this. It should be noted however, that Becker et al. [29], Kralj et al. [20], as well as the present study rely on relatively small sample sizes. Tracking of more individuals, ideally in several colonies around Europe would help to further investigate these strategies and prove the existence of different patterns for birds using different wintering areas.

Ecological implications:

The coexistence of divergent migratory strategies in birds of the Riether Werder colony may be a result of its location within the overlapping area of western European Common Terns using the western route [8, 15, 16, 26] and eastern European Terns using the eastern route [47]. This co-existence may have important ecological and evolutionary implications, even in the absence of assortative mating [25]. Divergent non-breeding distributions likely expose individuals to contrasting environmental conditions, generating carry-over effects on physiological condition, phenology, and general fitness that do not translate into genetic differentiation but nonetheless may influence the individuals breeding performance. Similar effects have been shown in House Martins (*Delichon urbicum*) breeding in a single colony but wintering in different African wintering areas [74]. Ecologically however, such within-colony variation may enhance population-level resilience by spreading risk across different routes and multiple wintering areas, lowering the risk of environmental change or anthropogenic pressures acting on a single migratory route or wintering area to affect the whole breeding population. This might prove beneficial to face current threats as the effects of climate change, already contributing to strong range shifts of prey species in the Benguela current upwelling system [75, 76] and altering monsoon circulation and marine food webs in the Indian ocean [77].

## Conclusion

We identified important stopover sites along both the eastern and western migration route of European Common Terns, as well as their main wintering areas. Our study aligns with a growing number of tracking studies in Europe, and hopefully will be useful to conserve habitats crucial for Common Terns to complete their annual cycle. Despite the growing numbers of tracking studies, so far the Riether Werder colony at the German Baltic coast is the only known place where birds using both migration routes and wintering in three distinct areas were documented to breed not only in a single colony but also with each other. This setup gives the perfect opportunity to investigate differences in the timing of migration, which reveals that previously reported differences between populations might not be dependent on the breeding population, but on the sex of migrating individuals, migratory route and wintering areas. Using different models, we identified the route and wintering area as the most important factor affecting duration of migration and stopovers, and sex as an additional factor effecting especially the timing of migration. The Riether Werder colony will be the perfect place for future studies on Common Tern migration, especially as the migratory phenology of a large number of birds is already known, and genetic samples have been already collected for most of the tracked birds.

## Electronic supplementary material

Below is the link to the electronic supplementary material.

Supplementary Material 1

Supplementary Material 2

Supplementary Material 3

Supplementary Material 4

## Data Availability

All data of timing and duration of migration is included in the additional file 2. Tracking data of all birds will be uploaded to the Movebank Data Repository upon acceptance of the manuscript.

## References

[1] Lack D. Bird migration and natural selection. Oikos. 1968;19(1):1–9. 10.2307/3564725.

[2] Alerstam T, Hedenström A, Åkesson S. Long-distance migration: evolution and determinants. Oikos. 2003;103:247–60.

[3] Hedenström A. Adaptations to migration in birds: behavioural strategies, morphology and scaling effects. Phil Trans R Soc B. 2008;363(1490):287–99. 10.1098/rstb.2007.2140. 17638691 10.1098/rstb.2007.2140 PMC2606751

[4] Piro S, Schmitz Ornés A. Revealing different migration strategies in a Baltic Common Tern (*Sterna hirundo*) population with light-level geolocators. J Ornithol. 2022;163(3):803–15. 10.1007/s10336-022-01986-1.

[5] Morrison JP. Books. Sci Am. 1987;256(3):26–33. 10.1038/scientificamerican0387-26.

[6] Ilina VO, Berdikulov BT, Lei F, Filimonov AN, Akentyeva YE, Song G, et al. Migration patterns and spatial connectivity of Pallas’s gulls (*ichthyaetus ichthyaetus*) from Alakol lake, Kazakhstan using ring recovery and tracking data. Avian res. 2025. 10.1016/j.avrs.2025.100253.

[7] Runge CA, Watson JEM, Butchart SHM, Hanson JO, Possingham HP, Fuller RA. Protected areas and global conservation of migratory birds. Science. 2015;350(6265):1255–58. 10.1126/science.aac9180. 26785490 10.1126/science.aac9180

[8] Becker PH, Ludwigs JD. Sterna hirundo common tern. In: Parkin D, editor. BWP update. Vol. 6. New York: Oxford University Press; 2004. p. 93–139.

[9] Elliott CCH. Analysis of the ringing and recoveries of three migrant terns. Ostrich. 1971;42(sup1):71–82. 10.1080/00306525.1971.9633398.

[10] Heinicke T, Herrmann C, Köppen U. Migration und Ansiedlungsverhalten ausgewählter Küstenvogelarten (Charadriidae, Laridae, Sternidae) in Mecklenburg-Vorpommern. Eine Auswertung von Ringfunden. Natur Naturschutz Mecklenb Vorpomm. 2016;44:3.

[11] Lundberg S, Alerstam T. Bird migration patterns: conditions for stable geographical population segregation. J. Theor. Biol. 1986;123(4):403–14. 10.1016/S0022-5193(86)80210-7.

[12] Salomonsen F. The evolutionary significance of bird-migration. Dan Biol Med. 1955;22:6.

[13] Muselet D. Les quartiers d’hivernage des Sternes pierregarins (*Sterna hirundo*) européennes. Oiseau. 1982;52:219–35.

[14] Radford MC. A study of the British ringing records of the common tern and arctic tern and comparison with some foreign records. Bird Study. 1961;8(4):174–84. 10.1080/00063656109476003.

[15] Fransson T, Österblom H, Hall-Karlsson S, Nikolopoulou S, Fransson T. Fuelling in front of the barrier-are there age based behavioral differences in garden warblers sylvia borin? PeerJ. 2014;2. Naturhistoriska Riksmuseet 2008.10.7717/peerj.319. 10.7717/peerj.319 PMC3970810 24711970

[16] Bairlein F, Dierschke J, Dierschke V, Salewski V, Geiter O, Hüppop K, et al. Atlas des Vogelzugs. AULA Verlag, Wiebelsheim 2014.

[17] Valkama J, Saurola P, Lehikoinen A, Lehikoinen E, Piha M, Sola P, et al. The Finnish bird migration atlas. Finnish Museum Nat Hist Ministry Environ. 2014;2.

[18] Hume R. The Common Tern. London: Hamlyn; 1993.

[19] Fiedler W, Geiter O, Ringfunde KU. herausgepickt Vogelw. 2013;51:131–36.

[20] Kralj J, Martinović M, Jurinović L, Szinai P, Sütő S, Preiszner B. Geolocator study reveals east African migration route of central European Common Terns. Avian Res. 2020;11(1). 10.1186/s40657-020-00191-z.

[21] Pavlinec Ž, Piro S, Schmitz Ornés A, Jurinović L, Barišić S, Ćiković D, et al. Influence of ocean primary production on the activity pattern of wintering Common Terns. J Ornithol. 2025;166(3):633–45. 10.1007/s10336-025-02271-7.

[22] Berthold P. Bird migration: a general survey. Oxford: Oxford University Press; 2001.

[23] Bogdanova MI, Daunt F, Newell M, Phillips RA, Harris MP, Wanless S. Seasonal interactions in the black-legged kittiwake, *Rissa tridactyla*: links between breeding performance and winter distribution. Proc Biol Sci B: Biol Sci. 2011;278(1717):2412–18. 10.1098/rspb.2010.2601. 10.1098/rspb.2010.2601 PMC3125632 21208952

[24] Briedis M, Bauer S. Migratory connectivity in the context of differential migration. Biol Lett. 2018;14(12):20180679. 10.1098/rsbl.2018.0679. 30958254 10.1098/rsbl.2018.0679 PMC6303517

[25] Piro S, Schmitz Ornés A. Testing for assortative mating based on migratory phenotypes in the Common Tern (*Sterna hirundo*). Avian Res. 2025;16(2):100230. 10.1016/j.avrs.2025.100230.

[26] Kürten N, Schmaljohann H, Bichet C, Haest B, Vedder O, González-Solís J, et al. High individual repeatability of the migratory behaviour of a long-distance migratory seabird. Mov Ecol. 2022;10(1). 10.1186/s40462-022-00303-y. 10.1186/s40462-022-00303-y PMC8817581 35123590

[27] Nisbet ICT, Szczys P, Mostello CS, Fox JW. Female Common Terns *Sterna hirundo* start autumn migration earlier than males. Seabirds. 2011;24:103–06. 10.61350/sbj.24.103.

[28] Nisbet ICT, Wingate B, Szczys P. Demographic consequences of a catastrophic event in the isolated population of Common Terns at Bermuda. Waterbirds. 2010;33(3):405–10. 10.1675/063.033.0319.

[29] Becker PH, Schmaljohann H, Riechert J, Wagenknecht G, Zajková Z, González-Solís J. Common Terns on the East Atlantic flyway: temporal–spatial distribution during the non-breeding period. J Ornithol. 2016;157(4):927–40. 10.1007/s10336-016-1346-2.

[30] Nisbet ICT, Mostello CS, Veit RR, Fox JW, Afanasyev V. Migrations and winter quarters of five common terns tracked using geolocators. Waterbirds. 2011;34(1):32–39. 10.1675/063.034.0104.

[31] Herrmann C. Jahresbericht der AG Küstenvogelschutz Mecklenburg-Vorpommern 2017. Seevögel. 2018;39:10–17.

[32] Lončar V, Kralj J, Stronen AV, Grgurević M, Pavlinec Ž, Jurinović L, et al. High genetic diversity yet weak population genetic structure in European common terns. Sci Rep. 2024;14(1). 10.1038/s41598-024-80614-9. 10.1038/s41598-024-80614-9 PMC11589120 39587288

[33] Griffiths R, Double MC, Orr K, Dawson RJG. A DNA test to sex most birds. Mol Ecol. 1998;7(8):1071–75. 10.1046/j.1365-294x.1998.00389.x. 9711866 10.1046/j.1365-294x.1998.00389.x

[34] R Core Team. R: a Language and Environment for Statistical Computing. 2020. https://www.R-project.org/%3E;.version 4.0.0. 12 May 2020. R Foundation for Statistical Computing, Vienna, Austria.

[35] Team R. Rstudio: integrated development for R. Rstudio, PBC Boston. 2020. http://www.rstudio.com/. 1 Nov 2020.

[36] Lisovski S, Bauer S, Briedis M, Davidson SC, Dhanjal-Adams KL, Hallworth MT, et al. Light-level geolocator analyses: a user’s guide. The J Anim Ecol. 2020;89(1):221–36. 10.1111/1365-2656.13036. 31190329 10.1111/1365-2656.13036

[37] Wotherspoon S, Sumner M, Lisovski S. R package BAStag: basic data processing for light based geolocation archival tags. GitHub repository. 2016. https://github.com/SWotherspoon/BAStag. 1. June 2020.

[38] Rakhimberdiev E, Winkler DW, Bridge ES, Seavy NE, Sheldon D, Piersma T, et al. A hidden Markov model for reconstructing animal paths from solar geolocation loggers using templates for light intensity. Mov Ecol. 2015;3(1). 10.1186/s40462-015-0062-5. 10.1186/s40462-015-0062-5 PMC4606513 26473033

[39] Lüdecke D, Shachar MS, Patil I, Waggoner P, Makowski D. Performance: an R package for assessment, comparison and Testing of Statistical models. JOSS. 2021;6(60):3139. 10.21105/joss.03139.

[40] R Core Team. R: a Language and Environment for Statistical Computing. 2025. https://www.R-project.org/%3E;.version 4.5.2. 19 Dec 2025. R Foundation for Statistical Computing, Vienna, Austria.

[41] Long JA. Jtools: analysis and presentation of social Scientific data. R package version 2.2.0, 2022. https://cran.r-project.org/package=jtools.

[42] Lenth R, Piaskowski J. Emmeans: estimated marginal Means, aka least-squares Means 2025. https://doi.org/10.32614/CRAN.package.emmeans R package version 2.0.0, https://CRAN.R-project.org/package=emmeans.

[43] Fox J, Weisberg S. An R Companion to applied regression. 3rd. Thousand Oaks, CA; 2019. https://www.john-fox.ca/Companion/index.html.

[44] Wickham H. ggplot2: elegant Graphics for data analysis. New York: Springer-Verlag; 2016.

[45] Auguie B. gridExtra: miscellaneous functions for “grid”. Graphics. 2017. https://doi.org/10.32614/CRAN.package.gridExtra; R package version 2.3, https://CRAN.R-project.org/package=gridExtra.

[46] Alerstam T, Bäckman J, Grönroos J, Olofsson P, Strandberg R, Sjöberg S. Migration of black terns *Chlidonias niger* and common terns *Sterna hirundo* between south Sweden and the Atlantic coast of Africa. J Avian Biol. 2025;2025(3). 10.1111/jav.03348.

[47] Kiat Y, Haviv E, Perlmann Y, Schekler I. Connectivity between breeding sites, wintering areas, and migration routes in Common Terns (Sterna hirundo) breeding in the western Palaearctic. Ibis (Lond 1859). 2026. 10.1111/ibi.70062.

[48] Wearne K, Underhill LG. Walvis Bay: a key wetland for waders and other coastal birds in southern Africa. Wader Study Group Bul. 2005;107:24–30.

[49] Fiedler W, Geiter O, Ringfunde HC. herausgepickt Vogelw. 2018;56:281–84.

[50] Fiedler W, Geiter O, Ringfunde HC. herausgepickt Vogelw. 2020;58:423–27.

[51] Aebischer NJ, Coulson JC, Colebrookl JM. Parallel long-term trends across four marine trophic levels and weather. Nature. 1990;347(6295):753–55. 10.1038/347753a0.

[52] Platt T, Fuentes-Yaco C, Frank KT. Spring algal bloom and larval fish survival. Nature. 2003;423(6938):398–99. 10.1038/423398b. 12761538 10.1038/423398b

[53] Helmke P, Romero O, Fischer G. Northwest African upwelling and its effect on offshore organic carbon export to the deep sea. Global Biogeochem Cycles. 2005;19(4). 10.1029/2004GB002265.

[54] Shannon LV, Field JG. Field JG are fish stocks food-limited in the southern Benguela pelagic ecosystem? Mar. Ecol. Prog. Ser. 1985;22:7–19. 10.3354/meps022007.

[55] Gittings JA, Raitsos DE, Racault MF, Brewin RJW, Pradhan Y, Sathyendranath S, et al. Seasonal phytoplankton blooms in the Gulf of Aden revealed by remote sensing. Remote Sens Environ. 2017;189:56–66. 10.1016/j.rse.2016.10.043.

[56] Raitsos DE, Yi X, Platt T, Racault MF, Brewin RJW, Pradhan Y, et al. Monsoon oscillations regulate fertility of the Red sea. Geophys Res Lett. 2015;42(3):855–62. 10.1002/2014GL062882.

[57] José YS, Penven P, Aumont O, Machu E, Moloney CL, Shillington F, et al. Suppressing and enhancing effects of mesoscale dynamics on biological production in the Mozambique channel. J Mar Syst. 2016;158:129–39. 10.1016/j.jmarsys.2016.02.003.

[58] Méndez V, Gill JA, Þórisson B, Vignisson SR, Gunnarsson TG, Alves JA. Paternal effects in the initiation of migratory behaviour in birds. Sci Rep. 2021;11(1). 10.1038/s41598-021-81274-9. 10.1038/s41598-021-81274-9 PMC7854704 33531548

[59] Neves VC, Nava CP, Cormons M, Bremer E, Castresana G, Lima P, Azevedojun SM, Phillips RA, Magalhaes MC, Santos RS. Migration routes and non-breeding areas of common terns (Sterna hirundo) from the Azores. Emu. 2015;115:158–67. . 10.1071/MU13112

[60] Ledwoń M, Neubauer G. Offspring desertion and parental care in the Whiskered Tern Chlidonias hybrida. Ibis (Lond 1859). 2017;159(4):860–72. 10.1111/ibi.12496.

[61] Byholm P, Beal M, Isaksson N, Lötberg U, Åkesson S. Paternal transmission of migration knowledge in a long-distance bird migrant. Nat Commun. 2022;13(1). 10.1038/s41467-022-29300-w. 10.1038/s41467-022-29300-w PMC8943069 35322030

[62] Arnqvist G, Rowe L. Sexual conflict. New York: Princeton University Press; 2005.

[63] Newton I. Migration within the annual cycle: species, sex and age differences. J Ornithol. 2011;152(S1):169–85. 10.1007/s10336-011-0689-y.

[64] Bond AL, Diamond AW. Nutrient allocation for egg production in six Atlantic seabirds. Can J Zool. 2010;88(11):1095–102. 10.1139/Z10-082.

[65] Meijer T, Drent R. Re-examination of the capital and income dichotomy in breeding birds. Ibis (Lond 1859). 1999;141(3):399–414. 10.1111/j.1474-919X.1999.tb04409.x.

[66] Reynolds CM. Mute swan weights in relation to breeding. Wildfowl. 1972;23:111–18.

[67] Drent R, Daan S. The prudent parent: energetic adjustments in avian breeding. Ardea. 2002;38-90:225–52. 10.5253/arde.v68.p225.

[68] Drent R. The timing of birds’ breeding seasons: the Perrins hypothesis revisited especially for migrants. Ardea. 2006;94:305–22.

[69] Williams CT, Klaassen M, Barnes BM, Buck CL, Arnold W, Giroud S, et al. Seasonal reproductive tactics: annual timing and the capital-to-income breeder continuum. Phil Trans R Soc B. 2017;372(1734):372. 10.1098/rstb.2016.0250. 10.1098/rstb.2016.0250 PMC5647277 28993494

[70] Bratton RM, Legett HD, Shannon P, Yakola KC, Gerson AR, Staudinger MD. Pre-breeding foraging ecology of three tern species nesting in the Gulf of Maine. ACE. 2022;17(1). 10.5751/ACE-02112-170119.

[71] Hobson KA, Sirois J, Gloutney ML. Tracing nutrient allocation to reproduction with stable isotopes: a preliminary investigation using colonial waterbirds of Great Slave Lake. The Auk. 2000;117(3):760–74. 10.1093/auk/117.3.760.

[72] Nilsson C, Klaassen RHG, Alerstam T. Differences in speed and duration of bird migration between spring and autumn. The Am Naturalist. 2013;181(6):837–45. 10.1086/670335. 23669545 10.1086/670335

[73] Liechti F. Birds: blowin’ by the wind? J Ornithol. 2006;147(2):202–11. 10.1007/s10336-006-0061-9.

[74] López-Calderón C, Hobson KA, Marzal A, Balbontín J, Reviriego M, Magallanes S, et al. Environmental conditions during winter predict age- and sex-specific differences in reproductive success of a trans-saharan migratory bird. Sci Rep. 2017;7(1). 10.1038/s41598-017-18497-2. 10.1038/s41598-017-18497-2 PMC5741763 29273801

[75] Crawford RJM, Makhado AB, Whittington PA, Randall RM, Oosthuizen WH, Waller LJ. A changing distribution of seabirds in south Africa the possible impact of climate and its consequences. Front. Ecol. Evol. 2015;3:10. 10.3389/fevo.2015.00010.

[76] Grémillet D, Peron C, Kato A, Amélineau F, Ropert-Coudert Y, Ryan PG, et al. Starving seabirds: unprofitable foraging and its fitness consequences in Cape gannets competing with fisheries in the Benguela upwelling ecosystem. Mar Biol. 2016;163(2):35. 10.1007/s00227-015-2798-2.

[77] Roxy MK, Ritika K, Terray P, Masson S. The curious case of Indian ocean Warming. J Educ Chang Clim. 2014;27(22):8501–09. 10.1175/JCLI-D-14-00471.1.

