# Supplementary material for: Tracking of adult males and females across a migratory divide: migration strategies of a western baltic common tern (Sterna hirundo) population
Source: Mov Ecol. 2026 May 30;14:37. doi: 10.1186/s40462-026-00666-6 (PMC13224715; doi:10.1186/s40462-026-00666-6)
Supplement: Supplementary file 1 — Supplementary Material 1 [file 40462_2026_666_MOESM1_ESM.docx]

**Additional file 1: Programming of loggers and analyses of logger data**

Loggers were programmed to sample light intensity and wet/dry data (mode 6b in the device settings). Light intensity was sampled every minute and the maximum light intensity was recorded every 5 minutes. The analyses of the light-level data stored in recovered loggers were conducted in R (v4.0.0, R Core Team 2020) using R Studio (v4.0.2, RStudio Team 2020) according to the online supplementary manual of Lisovski et al. (2020).The packag BAStag (v0.1.3 Wotherspoon et al. 2016) was used for twilight determination and FLightR (v0.5.1 Rakhimberdiev et al. 2015) for the analyses. Twilight determination was done with a light-level threshold of 1.5 lux. Extreme outliers (caused by periods of strong shading at day or artificial lighting at night) were removed manually. Data recorded during the breeding time were excluded from the analyses. Two 5–20-day calibration periods at the breeding site were set individually for each bird (with full track). One after the incubation period and prior to migration, second after returning to the colony and prior to incubation. Second calibration was not possible in birds with incomplete tracks.

Based on a preliminary and unconstrained analysis of the geolocation data using the software Intiproc, (v1.03, provided by Migrate Technology), the final model was constrained between 35° W, 40° S, 25° E and 60° N for birds using the western migration route, and 5° W, 40° S, 70° E and 60° N for birds using the eastern migration route, using the make.grid function. The model was run with the 1e6 particle filter recommended by Lisovski et al. (2020).Stopover analyses were conducted with a cut-off probability (minimal threshold probability of moving, Lisovski et al. 2020) of 0.4.

The full R-script used for the analyses can be found in Online Resource 1 of Piro and Schmitz Ornés (2022).

Literature used for the R-script of logger analysis:

Lisovski S, Bauer S, Briedis M, Davidson SC, Dhanjal-Adams KL, Hallworth MT, Karagicheva J, Meier CM, Merkel B, Ouwehand J, Pedersen L, Rakhimberdiev E, Roberto-Charron A, Seavy NE, Summer MD, Taylor CM, Wotherspoon SJ, Bridge ES. Light‐level geolocator analyses: A user's guide. J. Anim. Ecol. 2020; https://doi.org/10.1111/1365-2656.13036.

Piro S, Schmitz Ornés A. Revealing different migration strategies in a Baltic Common Tern (Sterna hirundo) population with light-level geolocators. J Ornithol 2022; https://doi.org/10.1007/s10336-022-01986-1

Rakhimberdiev E, Winkler DW, Bridge ES, Seavy NE, Sheldon D, Piersma T, Saveliev A. A hidden Markov model for reconstructing animal paths from solar geolocation loggers using templates for light intensity. Mov Ecol 2015; https://doi.org/10.1186/s40462-015-0062-5

R Core Team. R: A Language and Environment for Statistical Computing. R Foundation for Statistical Computing, Vienna, Austria. 2020; https://www.R-project.org/>.version 4.0.0. Accessed 12 May 2020.

RStudio Team. RStudio: Integrated Development for R. RStudio, PBC, Boston. 2020; http://www.rstudio.com/. Accessed 01 Nov 2020.

Wotherspoon S, Sumner M, Lisovski S. R Package BAStag: Basic data processing for light based geolocation archival tags. GitHub Repository 2016; https://github.com/SWotherspoon/BAStag. Accessed 01. June 2020.
