# Supplementary material for: Tracking of adult males and females across a migratory divide: migration strategies of a western baltic common tern (Sterna hirundo) population
Source: Mov Ecol. 2026 May 30;14:37. doi: 10.1186/s40462-026-00666-6 (PMC13224715; doi:10.1186/s40462-026-00666-6)
Supplement: Supplementary file 2 — Supplementary Material 2 [file 40462_2026_666_MOESM2_ESM.docx]

**Additional file 2: Summary of timing and duration of migration of all birds (73 tracks of 71 individuals as NA028195 and NA121822 were tracked twice)**

| **Ringnr.** | **Loggernr.** | **Sex** | **Migration route** | **Wintering area** | **Start autumn migration** | **Arrival wintering area** | **Duration autumn migration (days)** | **Days at stopover sites** | **Start spring migration** | **Arrival breeding area** | **Duration spring migration (days)** | **Days at stopover sites** |
| --- | --- | --- | --- | --- | --- | --- | --- | --- | --- | --- | --- | --- |
| NA028158 | CG170 | na | western | south | 09.08.2022 | 04.09.2022 | 27 | 7 | 21.03.2023 | 23.04.2023 | 34 | 24 |
| NA028195 | CG166 | male | western | south | 03.08.2022 | 03.09.2022 | 32 | 7 | 13.03.2023 | 18.04.2023 | 37 | 14 |
| *NA028195* | *CG166* | *male* | *western* | *south* | *15.08.2023* | *26.09.2023* | *43* | *26* | *13.03.2024* | *15.04.2024* | *34* | *0* |
| NA038608 | CG155 | female | western | west | 22.08.2022 | 09.09.2022 | 19 | 6 | 19.03.2023 | 25.04.2023 | 38 | 30 |
| NA038626 | BS686 | na | western | south | 08.09.2020 | 16.10.2020 | 39 | 23 | 02.04.2021 | 23.04.2021 | 22 | 6 |
| NA038659 | BS697 | male | western | south | 18.08.2019 | 18.09.2019 | 32 | 15 | 24.03.2020 | 23.04.2020 | 31 | 18 |
| NA038698 | BS694 | male | western | west | 01.08.2020 | 21.08.2020 | 22 | 14 | 18.03.2021 | 19.04.2021 | 33 | 21 |
| NA038700 | CG149 | female | western | south | 17.07.2022 | 17.08.2022 | 32 | 10 | 05.03.2023 | 13.04.2023 | 40 | 29 |
| NA090002 | BS685 | female | western | south | 31.08.2019 | 28.09.2019 | 28 | 0 | 20.03.2020 | 09.05.2020 | 51 | 38 |
| NA098476 | CG172 | female | western | south | 21.08.2022 | 02.10.2022 | 43 | 21 | 26.03.2023 | 24.04.2023 | 20 | 9 |
| NA108996 | CG165 | male | western | south | 03.09.2022 | 19.10.2022 | 47 | 23 | 21.03.2023 | 24.04.1934 | 35 | 19 |
| NA121680 | CG177 | male | western | west | 26.08.2022 | 02.10.2022 | 38 | 28 | 19.03.2023 | 22.04.2023 | 35 | 26 |
| NA121822 | CG151 | male | western | south | 25.08.2022 | 01.10.2022 | 38 | 20 | 24.03.2023 | 22.04.2023 | 30 | 9 |
| *NA121822* | *CG151* | *male* | *western* | *south* | *02.09.2023* | *06.10.2023* | *35* | *16* | *21.03.2024* | *19.04.2024* | *30* | *16* |
| NA132202 | BS684 | female | eastern | east | 26.07.2019 | 07.10.2019 | 73 | 60 | 29.02.2020 | 21.04.2020 | 53 | 42 |
| NA132210 | CG168 | female | eastern | east | 10.09.2022 | 07.11.2022 | 59 | 44 | 20.03.2023 | 21.04.2023 | 33 | 5 |
| NA132213 | BS673 | na | western | south | 03.08.2020 | 01.09.2020 | 30 | 9 | 17.03.2021 | 20.04.2021 | 35 | 16 |
| NA132217 | CG171 | female | eastern | east | 28.08.2022 | 14.11.2022 | 79 | 63 | 19.03.2023 | 23.04.2023 | 36 | 27 |
| NA132219 | BS673 | female | western | south | 18.07.2019 | 16.08.2019 | 30 | 6 | 05.03.2020 | 18.04.2020 | 40 | 19 |
| NA132224 | BS662 | female | eastern | east | 25.07.2019 | 17.10.2019 | 85 | 79 | 24.02.2020 | 22.04.2020 | 59 | 39 |
| NA132296 | BS679 | male | western | south | 08.09.2019 | 03.10.2019 | 26 | 11 | 22.03.2020 | 23.04.2020 | 34 | 17 |
| NA132313 | BS688 | male | western | south | 22.08.2019 | 08.10.2019 | 48 | 26 | 25.03.2020 | 23.04.2020 | 29 | 12 |
| NA132326 | CG141 | female | western | south | 30.08.2022 | 09.10.2022 | 41 | 21 | 19.03.2023 | 23.04.2023 | 36 | 18 |
| NA132332 | CG146 | male | western | south | 06.09.2022 | 07.11.2022 | 63 | 46 | 23.03.2023 | 24.04.2023 | 33 | 8 |
| NA132354 | CL787 | male | western | west | 13.09.2023 | 15.10.2023 | 33 | 25 | 26.03.2024 | 09.04.2024 | 15 | 0 |
| NA132359 | CG142 | male | western | south | 07.09.2022 | 13.11.2022 | 68 | 53 | 16.03.2023 | 23.04.2023 | 33 | 18 |
| NA132377 | BS688 | na | western | south | 10.09.2020 | 21.10.2020 | 42 | 31 | 19.03.2021 | 14.04.2021 | 27 | 12 |
| NA132379 | BS685 | male | eastern | east | 04.08.2020 | 15.09.2020 | 43 | 32 | 20.03.2021 | 30.04.2021 | 42 | 33 |
| NA160332 | CL796 | na | western | south | 03.09.2023 | 26.12.2023 | 115 | 89 | na | na | na | na |
| NA160339 | BS659 | female | western | south | 03.09.2020 | 05.10.2020 | 29 | 13 | 21.03.2021 | 22.04.2021 | 33 | 21 |
| NA161008 | BS684 | na | western | south | 06.08.2020 | 07.09.2020 | 33 | 23 | 14.03.2021 | na | na | na |
| NA163121 | BS675 | male | western | west | 08.09.2020 | 02.10.2020 | 25 | 15 | 03.04.2021 | 26.04.2021 | 24 | 17 |
| NA163137 | BS675 | male | western | south | 09.08.2019 | 30.09.2019 | 53 | 27 | 11.03.2020 | 10.04.2020 | 31 | 16 |
| NA163168 | BS661 | female | western | south | 07.08.2019 | 18.09.2019 | 43 | 22 | 11.03.2020 | 24.04.2020 | 45 | 27 |
| NA163172 | BS676 | female | western | south | 29.08.2019 | 21.09.2019 | 24 | 8 | 12.03.2020 | 23.04.2020 | 42 | 29 |
| NA163174 | BS698 | na | eastern | east | 19.08.2020 | 15.09.2020 | 28 | 19 | 19.03.2021 | 02.05.2021 | 45 | 24 |
| NA163175 | BS671 | male | western | south | 20.08.2019 | 20.09.2019 | 32 | 12 | 28.03.2020 | 24.04.2020 | 28 | 15 |
| NA163202 | BS681 | na | western | south | 22.08.2020 | 23.09.2020 | 33 | 16 | 19.03.2021 | 30.04.2021 | 43 | 20 |
| NA163205 | BS678 | female | western | south | 04.09.2019 | 29.09.2019 | 26 | 5 | 15.03.2020 | 21.04.2020 | 37 | 28 |
| NA163229 | BS666 | na | eastern | east | 21.07.2019 | 20.10.2019 | 91 | 81 | na | na | na | na |
| NA163230 | BS664 | female | western | south | 05.07.2019 | 16.08.2019 | 43 | 11 | 17.03.2020 | 29.04.2020 | 44 | 24 |
| NA163236 | CG138 | female | eastern | east | 24.08.2022 | 07.11.2022 | 76 | 57 | 08.03.2023 | 18.04.2023 | 42 | 27 |
| NA163247 | BS678 | female | eastern | east | 28.07.2020 | 11.12.2020 | 137 | 130 | 24.03.2021 | 23.04.2021 | 31 | 20 |
| NA163262 | BS661 | na | western | south | 06.08.2020 | 09.11.2020 | 96 | 7 | 14.03.2021 | 24.04.2021 | 42 | 14 |
| NA163414 | BS695 | female | eastern | south | 14.07.2019 | 01.11.2019 | 111 | 99 | 01.03.2020 | 19.04.2020 | 50 | 32 |
| NA163426 | CG150 | male | western | south | 30.07.2022 | 28.08.2022 | 30 | 16 | 28.03.2023 | 23.04.2023 | 27 | 19 |
| NA163439 | BS674 | male | eastern | east | 01.09.2019 | 01.10.2019 | 31 | 12 | 13.03.2020 | 21.04.2020 | 40 | 30 |
| NA163466 | CG153 | male | western | south | 25.08.2022 | 14.10.2022 | 51 | 33 | 23.03.2023 | 23.04.2023 | 32 | 16 |
| NA163470 | BS674 | male | western | west | 02.09.2020 | 04.10.2020 | 33 | 5 | 02.03.2021 | 03.05.2021 | 63 | 47 |
| NA163477 | BS662 | female | western | south | 17.07.2020 | 06.08.2020 | 21 | 0 | 20.03.2021 | 20.04.2021 | 32 | 17 |
| NA171504 | BS667 | male | western | west | 21.08.2019 | 28.08.2019 | 8 | 0 | 14.03.2020 | 24.04.2020 | 42 | 18 |
| NA171511 | CG157 | male | western | south | 26.08.2022 | 25.09.2022 | 31 | 17 | 21.03.2023 | 22.04.2023 | 33 | 20 |
| NA171512 | CL790 | male | western | south | 14.08.2023 | 25.09.2023 | 43 | 26 | 17.03.2024 | 10.04.2024 | 25 | 16 |
| NA171519 | BS694 | male | western | west | 31.08.2019 | 11.09.2019 | 12 | 0 | 25.03.2020 | 22.04.2020 | 29 | 24 |
| NA171564 | CL795 | female | western | east | 19.08.2023 | 04.10.2023 | 47 | 30 | 12.03.2024 | 24.04.2024 | 44 | 12 |
| NA171574 | BS668 | female | western | south | 30.08.2020 | 25.09.2020 | 27 | 10 | 28.02.2021 | 13.04.2021 | 46 | 33 |
| NA171575 | BS692 | female | western | south | 10.08.2019 | 26.09.2019 | 48 | 31 | 14.03.2020 | 17.04.2020 | 35 | 16 |
| NA171576 | CG148 | male | western | west | 11.08.2022 | 01.09.2022 | 22 | 0 | 06.03.2023 | 18.04.2023 | 44 | 25 |
| NA171631 | CG175 | female | western | na | 30.08.2022 | na | na | na | na | na | na | na |
| NA171636 | BS686 | female | western | south | 16.08.2019 | 21.09.2019 | 37 | 16 | 14.03.2020 | 27.04.2020 | 45 | 16 |
| NA171637 | BS681 | male | eastern | east | 22.08.2019 | 05.09.2019 | 15 | 6 | 28.02.2020 | 22.04.2020 | 55 | 44 |
| NA172931 | BS668 | male | western | south | 20.09.2019 | 19.10.2019 | 30 | 20 | 30.03.2020 | 25.04.2020 | 27 | 14 |
| NA172966 | BS671 | male | western | west | 14.08.2020 | 02.09.2020 | 20 | 12 | 24.03.2021 | 20.04.2021 | 28 | 20 |
| NA172971 | CG163 | male | western | south | 03.09.2022 | 19.10.2022 | 47 | 28 | 14.03.2023 | 24.04.2023 | 42 | 14 |
| NA172974 | BS698 | female | western | south | 21.08.2019 | 27.09.2019 | 38 | 16 | 14.03.2020 | 13.04.2020 | 31 | 12 |
| NA176437 | CG176 | female | western | south | 29.08.2022 | 28.09.2022 | 31 | 15 | 17.03.2023 | 25.04.2023 | 40 | 19 |
| NA176466 | CG143 | female | western | south | 28.07.2022 | 25.08.2023 | 29 | 6 | 23.03.2023 | 29.04.2023 | 38 | 28 |
| NA176467 | CG152 | female | western | na | 26.07.2022 | na | na | na | na | na | na | na |
| NA190054 | BS659 | male | eastern | east | 30.07.2019 | 08.10.2019 | 71 | 52 | 08.03.2020 | 18.04.2020 | 42 | 17 |
| NA190056 | BS687 | male | western | south | 03.09.2019 | 04.10.2019 | 32 | 17 | 05.04.2020 | 02.05.2020 | 28 | 18 |
| NA190118 | CG159 | female | western | west | 26.08.2022 | 10.09.2022 | 17 | 8 | 21.03.2023 | 22.04.2023 | 33 | 11 |
| NA190280 | BS697 | na | western | south | 05.08.2020 | 22.11.2020 | 110 | 86 | 18.03.2021 | 18.04.2021 | 32 | 16 |
| NA204734 | BS676 | na | western | south | 31.07.2020 | 04.09.2020 | 36 | 23 | 15.03.2021 | 28.04.2021 | 45 | 22 |
