# Supplementary material for: Tracking of adult males and females across a migratory divide: migration strategies of a western baltic common tern (Sterna hirundo) population
Source: Mov Ecol. 2026 May 30;14:37. doi: 10.1186/s40462-026-00666-6 (PMC13224715; doi:10.1186/s40462-026-00666-6)
Supplement: Supplementary file 3 — Supplementary Material 3 [file 40462_2026_666_MOESM3_ESM.docx]

**Additional file 3: Results (including effect plots) of the best linear models conducted to test the influence of sex, migration cycle (years), and wintering area and route (collapsed into one variable) on the migration of common terns breeding in North Eastern Germany.**

**Number of trackings included in the analyses:** 55 after eliminating those with unknown sex or wintering area and double tracking.

Here we present the best combination of variables for each model (response variable) using the *step* function for a backward stepwise regression in R (R Core Team 2025). Additionally, function *summ* was used from the package jtools (Long 2022) to create the model-summary table. Pairwise contrast was done using the function *emmeans* of the package “emmeans” (Lenth et al 2025), making p value adjustments with the tukey method for comparing a family of 3 or 4 estimates. We added a table comparing a null model with the full and best models using the function *compare_performances* from the “performance” package (Lüdecke et al 2021).

RESPONSE VARIABLES

- Start of autumn migration: “start_a"
- Duration of autumn migration: "duration_a"
- Stopover time during autumn migration: "stopover_a"
- Arrival in the wintering areas: "arrival_w"
- Start of spring migration: “start_s"
- Duration of spring migration: "duration_s"
- Stopover time during spring migration: "stopover_s"
- Arrival in the breeding areas: "arrival_B"

EXPLANATORY VARIABLES: The models included 3 factors in each case:

- Sex: ”sex”
  - males “*M*” (31 individuals) and females “*F*” (27 individuals)
- Migration cycle (years): “migration.cycle”
  - 2019_2020, 2020_2021, 2022_2023, and 2023_2024
- Wintering area and route (collapsed into one variable): “WBQRoute”
  - Using the eastern route and wintering in east: *e.eastern* (10 individuals),
  - Using the western route and wintering in south: s.western (35 individuals),
  - Using the western route and wintering in west: w.western (11 individuals)

Note: There were two more levels not considered in the models due to the low sample size. These results will be discussed outside of the model:

- - Using the eastern route and wintering in south: *s.eastern* (1 individual)
  - Using the western route and wintering in east: *e.western* (1 individual)

**Start of autumn migration:**

**Best model:**

Observations: 55

Dependent Variable: start_a

Type: OLS linear regression

MODEL FIT:

F(1,53) = 5.301939, p = 0.025258

R² = 0.090939

Adj. R² = 0.073787

Standard errors: Robust, type = HC1

--------------------------------------------------------------------------------------------------------------------------------

Est. S.E. t val. p

--------------------------------------------------------------------------------------------------------------------------------

(Intercept) 226.160000 3.803612 59.459269 0.000000

sexM 10.173333 4.548377 2.236695 0.029537

--------------------------------------------------------------------------------------------------------------------------------

**Model Comparison**:

-----------------------------------------------------------------------------------------------------------------------------------------------------

Model AIC (weights) AICc (weights) BIC (weights) R2 R2 (adj.) RMSE Sigma Delta_AIC

-----------------------------------------------------------------------------------------------------------------------------------------------------

Best start_a 467.2 (0.815) 467.6 (0.813) 473.2 (0.650) 0.091 0.074 16.016 16.315 0.000

Null start_a 470.4 (0.161) 470.7 (0.181) 474.4 (0.350) 0.000 0.000 16.798 16.953 3.244

Full start_a 474.2 (0.025) 477.3 (0.006) 490.2 (<.001) 0.139 0.031 15.586 16.684 7.008

-----------------------------------------------------------------------------------------------------------------------------------------------------

- **Duration of autumn migration:**

**Best model:**

Observations: 55

Dependent Variable: duration_a

Type: OLS linear regression

MODEL FIT:

F(2,52) = 18.623029, p = 0.000001

R² = 0.417341

Adj. R² = 0.394931

Standard errors: Robust, type = HC1

--------------------------------------------------------------------------------------------------------------------------------

Est. S.E. t val. p

--------------------------------------------------------------------------------------------------------------------------------

(Intercept) 22.636364 2.704156 8.370953 0.000000

WQBRoutes.western 15.040107 3.314025 4.538320 0.000034

WQBRoutee.eastern 44.263636 10.703830 4.135308 0.000130

--------------------------------------------------------------------------------------------------------------------------------

contrast estimate SE df t.ratio p.value

w.western - s.western -15.0 5.89 52 -2.554 0.0357

w.western - e.eastern -44.3 7.42 52 -5.967 <.0001

s.western - e.eastern -29.2 6.11 52 -4.785 <.0001

**Model Comparison**:

-----------------------------------------------------------------------------------------------------------------------------------------------------

Model AIC (weights) AICc (weights) BIC (weights) R2 R2 (adj.) RMSE Sigma Delta_AIC

---------------------------------------------------------------------------------------------------------------------------------

Best duration_a 472.5 (0.853) 473.3 (0.949) 480.5 (0.997) 0.417 0.395 16.508 16.978 0.000

Full duration_a 4076.0 (0.147) 479.2 (0.051) 492.1 (0.003) 0.463 0.396 15.850 16.966 3.523

Null duration_a 498.2 (<.001) 498.4 (<.001) 502.2 (<.001) 0.000 0.000 21.627 21.826 25.708

-----------------------------------------------------------------------------------------------------------------------------------------------------

- **Stopover time during autumn migration:**

**Best model:**

Observations: 55

Dependent Variable: stopover_a

Type: OLS linear regression

MODEL FIT:

F(2,52) = 18.657049, p = 0.000001

R² = 0.417785

Adj. R² = 0.395392

Standard errors: Robust, type = HC1

--------------------------------------------------------------------------------------------------------------------------------

Est. S.E. t val. p

--------------------------------------------------------------------------------------------------------------------------------

(Intercept) 10.272727 2.863720 3.587197 0.000739

WQBRoutes.western 8.050802 3.491885 2.305575 0.025156

WQBRoutee.eastern 43.227273 11.245274 3.844039 0.000331

--------------------------------------------------------------------------------------------------------------------------------

contrast estimate SE df t.ratio p.value

w.western - s.western -8.05 6.18 52 -1.304 0.3996

w.western - e.eastern -43.23 7.78 52 -5.557 <.0001

s.western - e.eastern -35.18 6.40 52 -5.492 <.0001

**Model Comparison**:

-----------------------------------------------------------------------------------------------------------------------------------------------------

Model AIC (weights) AICc (weights) BIC (weights) R2 R2 (adj.) RMSE Sigma Delta_AIC

-----------------------------------------------------------------------------------------------------------------------------------------------------

Best stopover_a 477.7 (0.835) 478.5 (0.942) 485.8 (0.996) 0.418 0.395 17.311 17.803 0.000

Full stopover_a 481.0 (0.165) 484.1 (0.058) 497.0 (0.004) 0.466 0.399 16.578 17.746 3.239

Null stopover_a 503.5 (<.001) 503.7 (<.001) 507.5 (<.001) 0.000 0.000 22.687 22.896 25.750

-----------------------------------------------------------------------------------------------------------------------------------------------------

- **Arrival time in the wintering area:**

**Best model:**

Observations: 55

Dependent Variable: arrival_w

Type: OLS linear regression

MODEL FIT:

F(2,52) = 6.001537, p = 0.004517

R² = 0.187539

Adj. R² = 0.156291

Standard errors: Robust, type = HC1

--------------------------------------------------------------------------------------------------------------------------------

Est. S.E. t val. p

--------------------------------------------------------------------------------------------------------------------------------

(Intercept) 258.181818 5.313293 48.591673 0.000000

WQBRoutes.western 10.465241 6.562470 1.594711 0.116838

WQBRoutee.eastern 33.618182 10.588815 3.174877 0.002519

--------------------------------------------------------------------------------------------------------------------------------

contrast estimate SE df t.ratio p.value

w.western - s.western -10.5 7.96 52 -1.315 0.3934

w.western - e.eastern -33.6 10.00 52 -3.353 0.0042

s.western - e.eastern -23.2 8.25 52 -2.805 0.0190

**Model Comparison**:

-----------------------------------------------------------------------------------------------------------------------------------------------------

Model AIC (weights) AICc (weights) BIC (weights) R2 R2 (adj.) RMSE Sigma Delta_AIC

-----------------------------------------------------------------------------------------------------------------------------------------------------

Best arrival_w 505.6 (0.673) 506.4 (0.850) 513.7 (0.840) 0.188 0.156 22.310 22.944 0.000

Full arrival_w 507.2 (0.311) 510.3 (0.123) 523.2 (0.007) 0.278 0.187 21.038 22.520 1.543

Null arrival_w 513.1 (0.016) 513.3 (0.028) 517.1 (0.153) 0.000 0.000 24.751 24.979 7.423

-----------------------------------------------------------------------------------------------------------------------------------------------------

- **Start of spring migration:**

**Best model:**

Observations: 55

Dependent Variable: start_s

Type: OLS linear regression

MODEL FIT:

F(3,51) = 4.008868, p = 0.012271

R² = 0.190818

Adj. R² = 0.143219

Standard errors: Robust, type = HC1

--------------------------------------------------------------------------------------------------------------------------------

Est. S.E. t val. p

--------------------------------------------------------------------------------------------------------------------------------

(Intercept) 74.431472 3.050055 24.403320 0.000000

sexM 4.583756 2.072332 2.211883 0.031481

WQBRoutes.western 1.247238 3.023510 0.412513 0.681692

WQBRoutee.eastern -6.164975 4.130756 -1.492457 0.141740

--------------------------------------------------------------------------------------------------------------------------------

contrast estimate SE df t.ratio p.value

w.western - s.western -1.25 2.88 51 -0.433 0.9020

w.western - e.eastern 6.16 3.64 51 1.694 0.2172

s.western - e.eastern 7.41 2.90 51 2.555 0.0358

**Model Comparison**:

-----------------------------------------------------------------------------------------------------------------------------------------------------

Model AIC (weights) AICc (weights) BIC (weights) R2 R2 (adj.) RMSE Sigma Delta_AIC

-----------------------------------------------------------------------------------------------------------------------------------------------------

Best start_s 391.2 (0.877) 392.4 (0.886) 401.2 (0.452) 0.191 0.143 7.741 8.039 0.000

Full start_s 396.2 (0.071) 399.4 (0.028) 412.3 (0.002) 0.205 0.106 7.673 8.214 5.029

Null start_s 396.9 (0.052) 397.1 (0.087) 400.9 (0.546) 0.000 0.000 8.606 8.685 5.645

-----------------------------------------------------------------------------------------------------------------------------------------------------

- **Duration of spring migration:**

**Best model:**

Observations: 55

Dependent Variable: duration_s

Type: OLS linear regression

MODEL FIT:

F(6,48) = 2.992559, p = 0.014505

R² = 0.272235

Adj. R² = 0.181264

Standard errors: Robust, type = HC1

-----------------------------------------------------------------------------------------------------------------------------------------------

Est. S.E. t val. p

-----------------------------------------------------------------------------------------------------------------------------------------------

(Intercept) 41.289682 3.379104 12.219121 0.000000

sexM -3.767598 2.019800 -1.865332 0.068253

WQBRoutes.western -2.031183 3.246192 -0.625713 0.534468

WQBRoutee.eastern 5.209486 4.103180 1.269621 0.210341

migration.cycle2020_2021 -2.788285 4.140894 -0.673353 0.503952

migration.cycle2022_2023 -3.781570 2.143794 -1.763961 0.084102

migration.cycle2023_2024 -9.998696 4.629682 -2.159694 0.035822

-----------------------------------------------------------------------------------------------------------------------------------------------

contrast estimate SE df t.ratio p.value

w.western - s.western 2.03 3.12 48 0.652 0.7924

w.western - e.eastern -5.21 3.81 48 -1.366 0.3666

s.western - e.eastern -7.24 3.00 48 -2.412 0.0508

contrast estimate SE df t.ratio p.value

2019_2020 - 2020_2021 2.788 3.41 48 0.817 0.8463

2019_2020 - 2022_2023 3.782 2.59 48 1.463 0.4676

2019_2020 - 2023_2024 9.999 4.62 48 2.166 0.1476

2020_2021 - 2022_2023 0.993 3.42 48 0.291 0.9913

2020_2021 - 2023_2024 7.210 5.18 48 1.392 0.5103

2022_2023 - 2023_2024 6.217 4.70 48 1.321 0.5540

**Model Comparison**:

-----------------------------------------------------------------------------------------------------------------------------------------------------

Model AIC (weights) AICc (weights) BIC (weights) R2 R2 (adj.) RMSE Sigma Delta_AIC

-----------------------------------------------------------------------------------------------------------------------------------------------------

Best/Full duration_s 396.3 (0.484) 399.5 (0.439) 412.4 (0.035) 0.272 0.181 7.680 8.221 0.000

Null duration_s 401.8 (0.031) 402.0 (0.121) 405.8 (0.930) 0.000 0.000 9.003 9.086 5.478

-----------------------------------------------------------------------------------------------------------------------------------------------------

- **Stopover time during spring migration:**

**Best model:**

Observations: 55

Dependent Variable: stopover_s

Type: OLS linear regression

MODEL FIT:

F(5,49) = 3.953763, p = 0.004322

R² = 0.287468

Adj. R² = 0.214760

Standard errors: Robust, type = HC1

-----------------------------------------------------------------------------------------------------------------------------------------------

Est. S.E. t val. p

-----------------------------------------------------------------------------------------------------------------------------------------------

(Intercept) 23.849941 3.142186 7.590238 0.000000

WQBRoutes.western -2.656208 3.066699 -0.866145 0.390632

WQBRoutee.eastern 5.390136 4.555700 1.183163 0.242453

migration.cycle2020_2021 1.282098 3.702111 0.346315 0.730588

migration.cycle2022_2023 -3.654989 2.562592 -1.426286 0.160126

migration.cycle2023_2024 -13.857785 4.923282 -2.814746 0.007010

-----------------------------------------------------------------------------------------------------------------------------------------------

contrast estimate SE df t.ratio p.value

w.western - s.western 2.66 3.19 49 0.833 0.6844

w.western - e.eastern -5.39 3.91 49 -1.378 0.3603

s.western - e.eastern -8.05 3.18 49 -2.530 0.0383

contrast estimate SE df t.ratio p.value

2019_2020 - 2020_2021 -1.28 3.61 49 -0.355 0.9845

2019_2020 - 2022_2023 3.65 2.73 49 1.338 0.5435

2019_2020 - 2023_2024 13.86 4.78 49 2.897 0.0278

2020_2021 - 2022_2023 4.94 3.62 49 1.363 0.5281

2020_2021 - 2023_2024 15.14 5.34 49 2.834 0.0326

2022_2023 - 2023_2024 10.20 4.82 49 2.117 0.1622

**Model Comparison**:

-----------------------------------------------------------------------------------------------------------------------------------------------------

Model AIC (weights) AICc (weights) BIC (weights) R2 R2 (adj.) RMSE Sigma Delta_AIC

-----------------------------------------------------------------------------------------------------------------------------------------------------

Best stopover_s 401.9 (0.581) 404.3 (0.655) 416.0 (0.306) 0.287 0.215 8.228 8.718 0.000

Full stopover_s 402.6 (0.412) 405.7 (0.319) 418.7 (0.079) 0.304 0.217 8.131 8.703 0.688

Null stopover_s 410.6 (0.008) 410.8 (0.026) 414.6 (0.615) 0.000 0.000 9.748 9.838 8.641

-----------------------------------------------------------------------------------------------------------------------------------------------------

- **Arrival time in the breeding area:**

**Best model:**

Observations: 55

Dependent Variable: arrival_B

Type: OLS linear regression

MODEL FIT:

F(3,51) = 3.915305, p = 0.013640

R² = 0.187198

Adj. R² = 0.139386

Standard errors: Robust, type = HC1

-----------------------------------------------------------------------------------------------------------------------------------------------

Est. S.E. t val. p

-----------------------------------------------------------------------------------------------------------------------------------------------

(Intercept) 113.652174 1.247362 91.114018 0.000000

migration.cycle2020_2021 -0.763285 2.335685 -0.326793 0.745162

migration.cycle2022_2023 -1.231121 1.466047 -0.839756 0.404965

migration.cycle2023_2024 -9.402174 2.433144 -3.864208 0.000316

-----------------------------------------------------------------------------------------------------------------------------------------------

contrast estimate SE df t.ratio p.value

2019_2020 - 2020_2021 0.763 2.00 51 0.382 0.9809

2019_2020 - 2022_2023 1.231 1.58 51 0.781 0.8628

2019_2020 - 2023_2024 9.402 2.76 51 3.412 0.0067

2020_2021 - 2022_2023 0.468 2.06 51 0.227 0.9958

2020_2021 - 2023_2024 8.639 3.06 51 2.826 0.0329

2022_2023 - 2023_2024 8.171 2.80 51 2.920 0.0259

**Model Comparison**:

-----------------------------------------------------------------------------------------------------------------------------------------------------

Model AIC (weights) AICc (weights) BIC (weights) R2 R2 (adj.) RMSE Sigma Delta_AIC

-----------------------------------------------------------------------------------------------------------------------------------------------------

Best arrival_B 340.9 (0.881) 342.1 (0.880) 350.9 (0.422) 0.187 0.139 4.898 5.086 0.000

Full arrival_B 346.2 (0.060) 349.4 (0.023) 362.3 (0.001) 0.196 0.096 4.871 5.214 5.386

Null arrival_B 346.3 (0.059) 346.5 (0.097) 350.3 (0.576) 0.000 0.000 5.433 5.483 5.400

-----------------------------------------------------------------------------------------------------------------------------------------------------
