# Supplementary material for: Tracking of adult males and females across a migratory divide: migration strategies of a western baltic common tern (Sterna hirundo) population
Source: Mov Ecol. 2026 May 30;14:37. doi: 10.1186/s40462-026-00666-6 (PMC13224715; doi:10.1186/s40462-026-00666-6)
Supplement: Supplementary file 4 — Supplementary Material 4 [file 40462_2026_666_MOESM4_ESM.docx]

**Additional file 4: Analyses of difference in duration of autumn and spring migration for 66 Common Terns tagged with light level geolocators in the Riether Werder colony.**

Number of tracks included in the analyses: 64 after eliminating those with incomplete tracing cycle or double tracking (only the 2023/2024 track used).

Wilcoxon signed rank tests and Holm-Bonferroni adjustment were used to compare the duration of autumn and spring migration.

For the separate analyses of birds using different routes and wintering in different areas birds were grouped:

- - Using the eastern route and wintering in east: *e.eastern* (11 individuals),
  - Using the western route and wintering in south: s.western (42 individuals),
  - Using the western route and wintering in west: w.western (11 individuals)

Note: There were two more levels not considered in the analyses due to the low sample size:

- - Using the eastern route and wintering in south: *s.eastern* (1 individual)
  - Using the western route and wintering in east: *e.western* (1 individual)

A Summary of the tests is found in Table S1; plots are shown in Fig. S1-S4.

**Table S1: Summary of analyses comparing the duration of autumn and spring migration for all birds as well as separated for the migration route and wintering area groups:**

| **Group** | **Duration of migration (mean ± sd)** | | **Wilcox-test** | ***p-*value** | **adjusted *p-*value  (Holm-Bonferroni)** |
| --- | --- | --- | --- | --- | --- |
|  | **Autumn** | **Spring** |  |  |  |
| all birds | 42.4 ± 24.5 days | 36.7 ± 9.0 days | V = 1281.5, n = 66 | 0.262 | 0.469 |
| e.eastern | 63.4 ± 33.9 days | 33.9 ± 9.0 days | V = 52.0 , n = 11 | 0.100 | 0.299 |
| w.western | 22.6 ± 9.1 days | 34.9 ± 12.4 days | V = 10.0, n = 11 | 0.045 | 0.181 |
| s.western | 40.3 ± 17.5 days | 34.9 ± 7.0 days | V = 547, n = 42 | 0.235 | 0.469 |


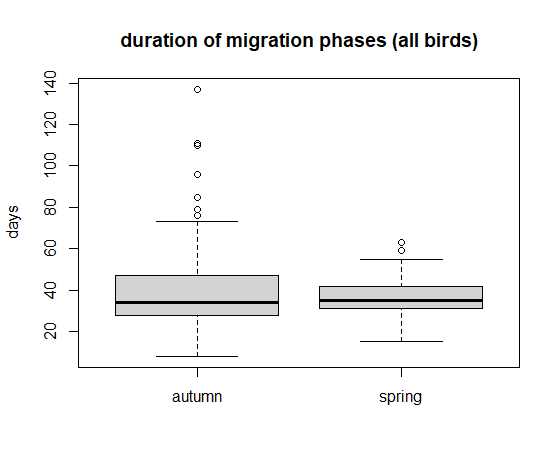


Figure S1: Comparison of duration of autumn and spring migration for all birds (n = 66), no significant difference found (Wilcox-test: V = 1281.5, *p* = 0.262, adjusted *p* = 0.469).


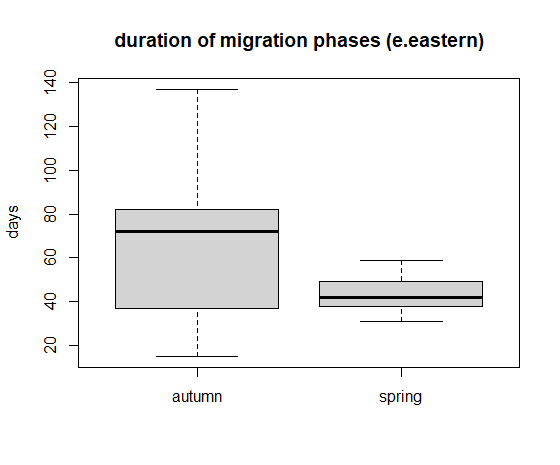


Figure S2: Comparison of duration of autumn and spring migration for birds using the eastern route and wintering in eastern Africa (n = 11), no significant difference found (Wilcox-test: V = 52, *p* = 0.100, adjusted *p* = 0.299).


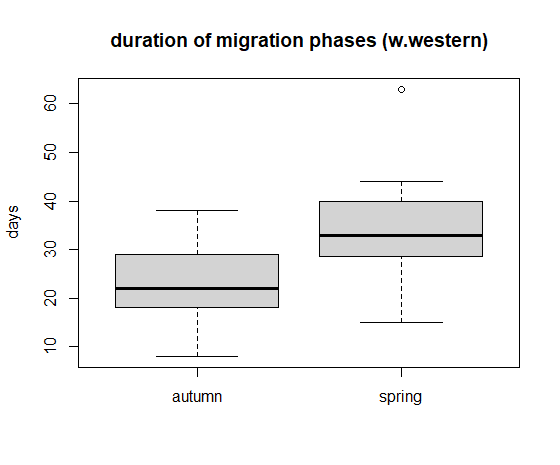


Figure S3: Comparison of duration of autumn and spring migration for birds using the western route and wintering in western Africa (n = 11), no significant difference found (Wilcox-test: V = 10, *p* = 0.045, adjusted *p* = 0.181).


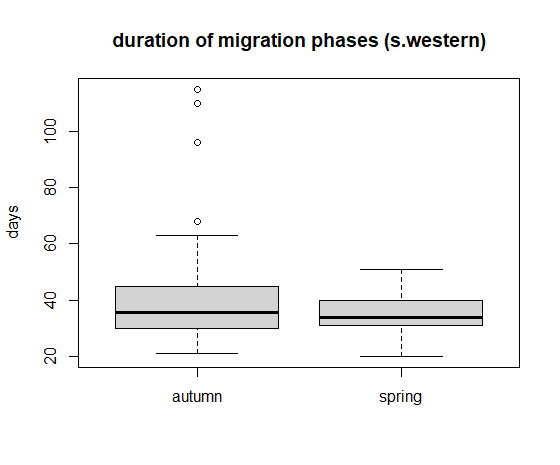


Figure S4: Comparison of duration of autumn and spring migration for birds using the western route and wintering in southern Africa (n = 42), no significant difference found (Wilcox-test: V = 547, *p* = 0.235, adjusted *p* = 0.469).
